# Plasma Kallikrein as a Modulator of Liver Injury/Remodeling

**DOI:** 10.3389/fphar.2021.715111

**Published:** 2021-09-09

**Authors:** Ibrahim A Ahmed, Miran A Jaffa, Mayssam Moussa, Duaa Hatem, Ghewa A El-Achkar, Rola Al Sayegh, Mia Karam, Eva Hamade, Aida Habib, Ayad A Jaffa

**Affiliations:** ^1^ Department of Biochemistry and Molecular Genetics, Faculty of Medicine, Beirut, Lebanon; ^2^ Epidemiology and Population Health Department, Faculty of Health Sciences, American University of Beirut, Beirut, Lebanon; ^3^ Section of Pharmacology, Department of Bioethics and Safety, Catholic University, Rome, Italy; ^4^ INSERM-UMR1149, Centre de Recherche sur l’Inflammation, and Sorbonne Paris Cité, Laboratoire d’Excellence Inflamex, Faculté de Médecine, Site Xavier Bichat, Universite de Paris, Paris, France; ^5^ Biomedical Engineering Program, Maroun Semaan Faculty of Engineering and Architecture, American University of Beirut, Beirut, Lebanon; ^6^ Laboratory of Cancer Biology and Molecular Immunology, Faculty of Sciences I, Lebanese University, Beirut, Lebanon

**Keywords:** plasma kallikrein, necrosis, inflammation, liver injury, kallikrein-kinin system, fibrosis, remodeling

## Abstract

The occurrence and persistence of hepatic injury which arises from cell death and inflammation result in liver disease. The processes that lead to liver injury progression and resolution are still not fully delineated. The plasma kallikrein-kinin system (PKKS) has been shown to play diverse functions in coagulation, tissue injury, and inflammation, but its role in liver injury has not been defined yet. In this study, we have characterized the role of the PKKS at various stages of liver injury in mice, as well as the direct effects of plasma kallikrein on human hepatocellular carcinoma cell line (HepG2). Histological, immunohistochemical, and gene expression analyses were utilized to assess cell injury on inflammatory and fibrotic factors. Acute liver injury triggered by carbon tetrachloride (CCl_4_) injection resulted in significant upregulation of the plasma kallikrein gene (Klkb1) and was highly associated with the high mobility group box 1 gene, the marker of cell death (*r* = 0.75, *p* < 0.0005, *n* = 7). In addition, increased protein expression of plasma kallikrein was observed as clusters around necrotic areas. Plasma kallikrein treatment significantly increased the proliferation of CCl_4_-induced HepG2 cells and induced a significant increase in the gene expression of the thrombin receptor (protease activated receptor-1), interleukin 1 beta, and lectin–galactose binding soluble 3 (galectin-3) (*p* < 0.05, *n* = 4). Temporal variations in the stages of liver fibrosis were associated with an increase in the mRNA levels of bradykinin receptors: beta 1 and 2 genes (*p* < 0.05; *n* = 3–10). In conclusion, these findings indicate that plasma kallikrein may play diverse roles in liver injury, inflammation, and fibrosis, and suggest that plasma kallikrein may be a target for intervention in the states of liver injury.

## Introduction

Liver injury is an eminent condition of the body system owing to the numerous functions of the liver. Hepatitis (Real et al., 2019), acute liver failure (Stravitz and Lee, 2019), cholestasis (Horvatits et al., 2019), nonalcoholic fatty liver disease (Singh et al., 2015), and alcoholic liver disease (Gao and Bataller, 2011) are the varying phenotypes resulting from acute and chronic liver injuries. Some of the hallmarks of these pathologies reside in the initiation of cell death (Wang, 2014), inflammatory (Zhangdi et al., 2019), and fibrotic mechanisms (Weiskirchen et al., 2018).

The coagulation system, a functional player of the cardiovascular system, is indicated as a driving force in liver injury and remodeling (Nault et al., 2016; Pant et al., 2018). This system modulates physiological and pathophysiological actions pertaining to neutrophil aggregation, vasodilation, inflammation, complement activation, and vascular tone (Ribeiro et al., 2014; Schmaier, 2016; Kenne et al., 2019). Previous studies of acute and chronic inflammation, and tissue remodeling have highlighted the role of the kallikrein-kinin system (KKS) (Ribeiro et al., 2014). The thrombin receptors, protease-activated receptors (PAR) 1 and 2 (Kallis et al., 2014; Nault et al., 2016; Shearer et al., 2016), and the bradykinin receptor, beta 2 (BDKRB2), are upregulated in liver fibrosis with a possible involvement of the latter and its ligand, bradykinin (BK) in fibrotic resolution (Sancho-Bru et al., 2007). However, other studies have shown that BDKRB2 is implicated in immune liver injury (Zhang et al., 2019), while the inhibition of the bradykinin receptor, beta 1 (BDKRB1), was shown to resolve the disorder (Zhang et al., 2018). Agonists of PARs like thrombin, tissue factor, trypsin, mast tryptase, coagulation factors Xa and VIIa, and plasma kallikrein have been studied over the past years (Gieseler et al., 2013; Heuberger and Schuepbach, 2019). Most compelling evidence stems from thrombin/factor Xa-induced PAR1 involvement in tissue fibrogenesis, while mast cell tryptase and factor Xa act through PAR2 to cause fibroblast proliferation, differentiation, and migration (Borensztajn et al., 2008; Borensztajn et al., 2010; Kitasato et al., 2014). In addition, experimental and human studies of acute and chronic liver disease showed the upregulation of thrombin, factor Xa and tissue factor, therefore implicating them as inducers of PARs in liver fibrogenesis and fibrosis progression (Marra et al., 1995; Pant et al., 2018). The cleavage of transforming growth factor, beta 1, the most potent fibrogenic factor, was recently described by plasma kallikrein, in hepatic stellate cells, and suppressing this mechanism by inhibition prevented acute liver injury (Li et al., 2018). This implicates plasma kallikrein as a driver of liver injury, yet more investigations are needed to ascertain this effect.

In this study, we explored the involvement of the PKKS in acute and chronic liver injury to unravel its possible roles in cell death, inflammation, and fibrosis. We applied correlation analysis to explore/establish relationships, risk, interactions, and possible involvement of some players as mediators of liver injury. Our findings demonstrated that components of the PKKS are associated with cell death, inflammation, myofibroblast activators, as well as fibrosis of liver injury, and may function as the indicators of oscillating molecular regulation in different phases of liver injury.

## Materials and Methods

### Animals Experimental Liver Injury

C57BL/6J male mice of 10–12 weeks old were used throughout the experiments and were obtained from the animal facility of the American University of Beirut. The mice were housed five per cage in a temperature- and humidity‐controlled room, kept on a 12‐hr light–dark cycle, and provided with food and water ad lib. All experimental procedures were approved and conducted following the guidelines of the Institutional Animal Care and Use Committee (IACUC: 19–08–541 and 19–08–542)

**FIGURE 1:**
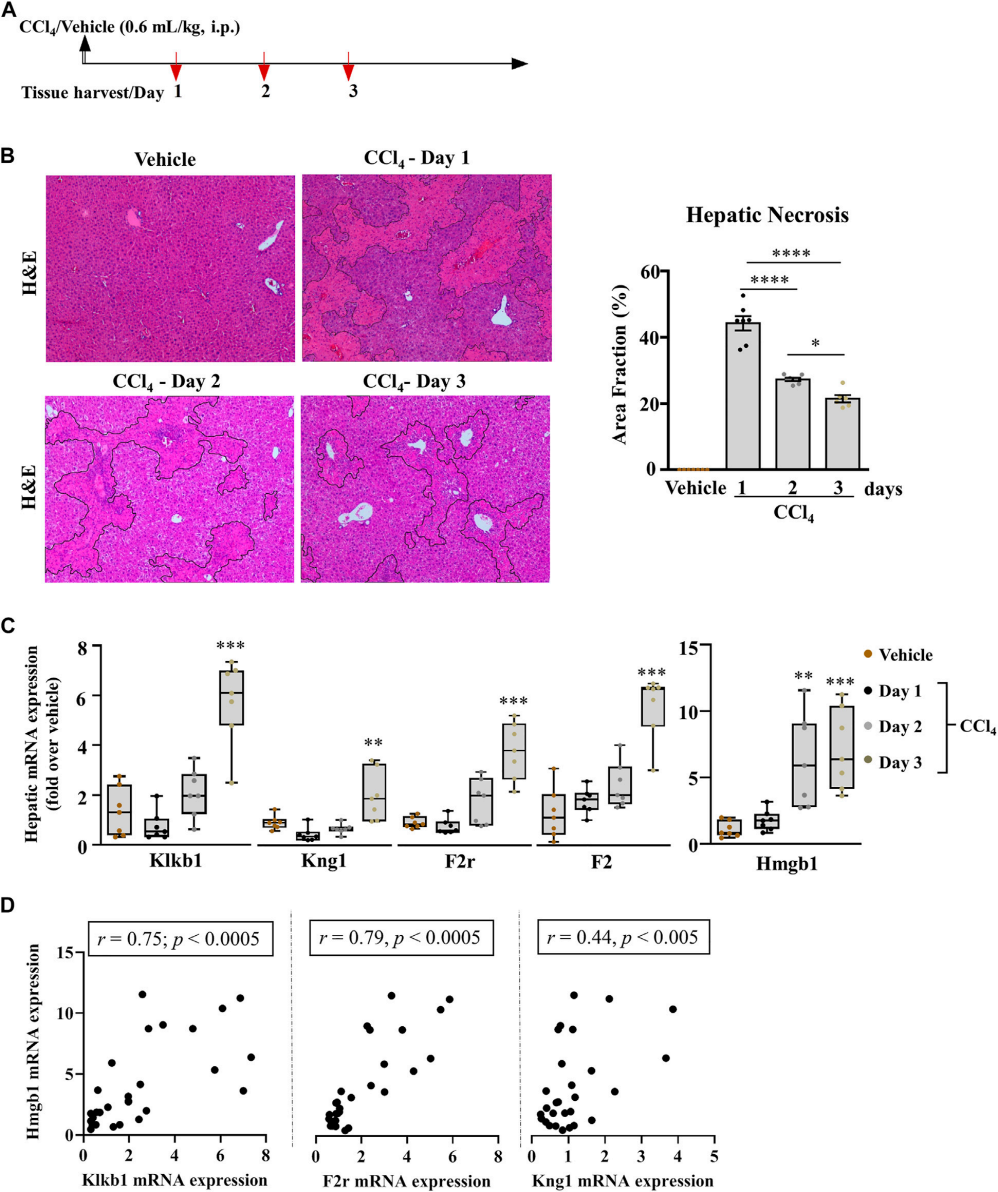
Pronounced modification in the hepatic PKKS genes in acutely injured mice. **(A)** Schematic representation of carbon tetrachloride (CCl_4_)-induced acute liver injury at days 1, 2, and 3 after the CCl_4_ and mineral oil (vehicle) injection. **(B)** Representative H& E (original magnification ×40) staining of liver sections; necrotic areas are delineated by marked areas quantified using ImageJ software; data are represented as mean ± SEM (one-way ANOVA followed by Sidak’s multiple comparisons, *p < 0.05, ****p <0.0001; *n* = 7 mice per group). **(C)** Gene expression analysis of the PKKS (Klkb1, Kng1, F2r, and F2) and Hmgb1 genes. Data are shown as minimum to maximum values of box plots with whiskers extending 1.5 times the interquartile range. Center lines indicate the medians, while box limits represent the 25th and 75th percentiles (*n* = 7 per group). Statistical significance was determined by one-way ANOVA followed by Sidak’s multiple comparisons, where **p <0.005, ***p <0.0005 (CCl_4_ vs. Vehicle). **(D)** Association between Hmgb1 and Klkb1, F2r or Kng1 genes, correlative plots were assessed by the Spearman correlation.

Acute liver injury: Acute liver injury was induced by a single intraperitoneal (i.p.) injection of 0.6 ml/kg carbon tetrachloride (CCl_4_) (270652 Sigma-Aldrich) diluted (1/10) in mineral oil (vehicle) (M5904 Sigma-Aldrich). The mice were sacrificed by cervical dislocation, and the liver tissues were harvested at each corresponding time point (Figure 1A).

Liver fibrosis: Chronic liver injury was induced by i.p. injection of diluted (1/10) 0.6 ml/kg CCl_4_ in mineral oil, twice a week for 2.5, 4, 6, or 7 weeks. Mice were sacrificed at the 1st or 3rd day after the last injection of CCl_4_.

### Histological and Immunohistochemical Experiments

Upon sacrifice, samples of each of the four liver lobes were cut and placed on microscopic slides. Cell death by necrosis was evaluated on hematoxylin and eosin (H&E), and collagen fiber deposition was carried out by the picro sirius red (PSR) staining. Immunohistochemistry **(**IHC) for plasma kallikrein was performed using a rabbit polyclonal antibody anti-plasma kallikrein (PA5-76711 Invitrogen), biotinylated goat anti-rabbit secondary antibodies were added for an hour while staining using 3,3’-diaminobenzidine (DAB), and counterstaining with hematoxylin (Leica Biosystem) was carried out as described previously (Habib et al., 2019). No staining was observed when omitting the primary antibody. Evaluation and quantification of necrosis and collagen fiber deposition were performed on photomicrographs of ten each per mouse, using ImageJ software (NIH, United States). Necrotic areas, identified by the absence of, or altered hepatic cells, were delineated and calculated to the total area of each photomicrograph. Collagen fibers were identified by their red deposition and quantified by utilizing ImageJ software.

### Hepatocellular Carcinoma Cell Line, Culture, and Treatment

Human hepatocellular carcinoma cell line, HepG2, was obtained from the ATCC (Virginia, United States). All necessary procedures from storage to culturing were performed according to the guidelines of the ATCC. The cells were cultured in low-glucose Dulbecco’s media (DMEM, D6046 Sigma-Aldrich) supplemented with 10% fetal bovine serum (FBS) and 1% penicillin-streptomycin (P/S), and incubated at 37°C. 1 or 2 mM CCl_4_ in 0.5% dimethyl sulfoxide (DMSO, 41640 Sigma-Aldrich), or 2.5 ng/ml plasma kallikrein (K2638 Sigma-Aldrich) were added to the cells. Viability and proliferation assays were conducted using a 96-well plate. HepG2 cells were plated in 1 × 10^5^ cells/well for viability assay (for cell death or toxicity determination) and 1 × 10^4^ cells/well for the proliferation assay. After the treatment of cells with CCl_4_ or/and plasma kallikrein for 24 h, viability and proliferation tests were measured using the MTT assay (M5655 Sigma-Aldrich) at an absorbance of 595 nm, and calculated according to the instruction of the manufacturer. 3 × 10^5^ HepG2 cells/well were plated in a 12-well plate for the plasma kallikrein study. RNA extraction and gene expression analysis were performed after the cells were treated with 2.5 ng/ml plasma kallikrein or media only (control groups) for 24 h.

### Gene Expression Profiles

Total RNA from the frozen liver tissue fragments obtained from the left and median lobes, homogenized in Qiazol Lysis Reagent (79306 Qiagen, Hilden, Germany), using a Tissue Lyser (QIAGEN II), was extracted as described previously (Habib et al., 2019). For HepG2 cells, the total RNA was extracted using TRIzol™ Reagent (15596026 Ambion Life Technologies). 2 µg of the total RNA were reverse-transcribed into cDNA using the High-Capacity Reverse Transcriptase kit (004007363 Thermo Fisher Scientific). Using the iTaq™ Universal SYBR Green Supermix (1725121 Bio-Rad Laboratories), real-time quantitative polymerase chain reactions (RT-qPCR) were performed in a CFX384 system (Bio-Rad Laboratories, California, United States). The primers (Macrogen Inc., Seoul, South Korea) were previously described (Habib et al., 2019), and others are listed in Supplementary Table 1. The results were calculated using the ΔΔCT method and normalized in the housekeeping genes 18S and GAPDH for liver tissues and HepG2 cells, respectively.

### Statistical Analysis

Statistical analysis was conducted using SPSS (Statistical Package for the Social Sciences) software and GraphPad Prism 8, (version 8.4.3 for Windows, GraphPad Software, La Jolla, CA 92037, United States). The test of normality was performed using the Shapiro test, while multiple comparisons between groups were conducted by one-way analysis of variances (ANOVA) followed by Sidak’s multiple comparison test or the Mann–Whitney U test; *p < 0.05, **p < 0.005, ***p < 0.0005, and ****p < 0.0001 are considered statistically significant. Correlation analysis was carried out by the Spearman correlation of nonparametric test, while the significance of the coefficient of correlation, *r*, was determined by *p* < 0.05.

## Results

### Genes for Plasma Kallikrein (Klkb1), High Molecular Weight Kininogen (Kng1), and PAR1 (F2r) Are Positively Associated to the Gene of High Mobility Group Box (Hmgb1), in Acute Liver Injury

We studied the role of the PKKS in acute liver injury of C57BL/6J mice treated with a single injection of 0.6 ml/kg of CCl_4_ i.p. Mice were sacrificed at day one, two, or three post CCl_4_ injection (Figure 1A). Necrosis was assessed by H&E staining on liver sections (Figure 1B delineated area) and showed a 45.1% increase in necrosis after day one of CCl_4_ treatment compared to the vehicle. A gradual decrease in this necrotic area was observed from days one to two, and two to three (39.3 and 22.9%, respectively, Figure 1B). Compared to vehicle-treated animals, injured liver presented a 42.5% decrease in hepatic mRNA expression of the Klkb1 gene at day one, and a 1.6- and 4.3-fold increase at days two and three, respectively (Figure 1C; *p* < 0.0005 at day three). Since plasma kallikrein activation modulates a high molecular weight kininogen (KNG1) (Moreau et al., 2005) and PAR1 signaling (Abdallah et al., 2010), we assessed their gene expression. Figure 1C shows a similar pattern of expression of 51.9 and 16.7% decrease 1 day after the last CCl_4_ injection in the hepatic mRNA levels of Kng1 and F2r. This suggests a modulation in the downstream target of plasma kallikrein. Since PAR1 has numerous ligands including plasma kallikrein, we assessed the thrombin gene (F2) expression in the liver, which showed an increased expression at day one injury, in contrast to the decrease depicted for Klkb1, Kng1, and F2r genes (Figure 1C). Next, we assessed the gene expression of high mobility group box (Hmgb1), a marker of cell death, which has been shown to have pro-inflammatory effects (Man et al., 2015; Paudel et al., 2018; Zhang et al., 2019). We observed a similar pattern of the expression of the Hmgb1 gene to the induced PKKS genes (Figure 1C). The Spearman correlation analysis showed a positive association between Hmgb1 gene and the PKKS genes (Klkb1, *r* = 0.75 *p <* 0.0005; F2r, *r* = 0.79; *p <* 0.0005; Kng1, *r* = 0.44, *p =* 0.019; Figure 1D, Supplementary Figures 1A,B).

### *In Vivo* and *In Vitro* Impacts of Plasma Kallikrein in Liver Injury

**FIGURE 2:**
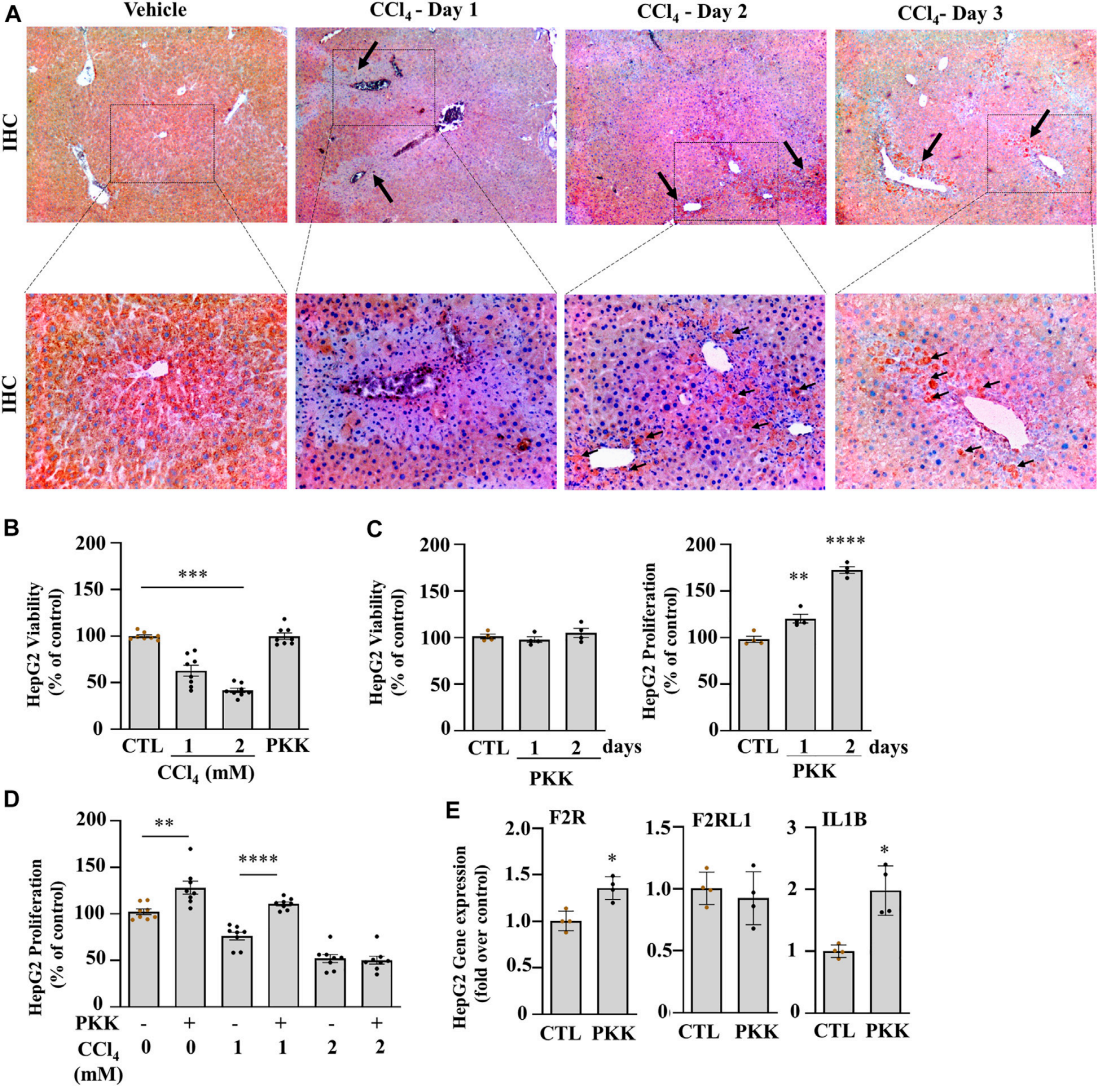
Klkb1 gene expression is translated to the pre-kallikrein protein and stimulates cell proliferation. **(A)** Representative images of immunostaining of plasma kallikrein (black arrows; dark red clusters; original magnification ×40 (top) and ×100 (bottom)) at days 1, 2, and 3 of liver injury after the CCl_4_ or vehicle (mineral oil) injection. **(B)** Percentage viability of HepG2 treated 1 or 2 mM CCl_4_ or 2.5 ng/ml plasma kallikrein (PKK) for 24 h (*n* = 8). **(C)** Percentage viability and **(D)** proliferation of plasma kallikrein–treated cells for 1 or 2 days (*n* = 8). Data are shown as mean ± SEM. Statistical significance was determined by one-way ANOVA followed by Sidak’s multiple comparisons, where *p < 0.05, **p < 0.005 (PKK vs. control,), ***p < 0.0005 (1 mM CCl_4_ vs. control; 2 mM CCl_4_ vs. 1mM CCl_4_), ****p < 0.0001 (Day 2-PKK vs. others; 1mM CCl_4_+PKK vs. 1mM CCl_4_). **(E)** Plasma kallikrein (PKK) increased the mRNA levels of F2R, and IL1B genes (*n* = 8). Data are shown as mean ± SEM. Statistical significance was determined by the Mann–Whitney test, *p < 0.05 (PKK versus CTL). CTL corresponds to the control group with 0.5 % DMSO as vehicle.

We further analyzed the expression of the plasma kallikrein protein by immunohistochemistry which showed a decrease on day 1 compared to the vehicle and formed clusters around the injured areas on day 2 and day 3 (Figure 2A; black arrows). To further investigate the role of plasma kallikrein in liver injury, we evaluated the effect of plasma kallikrein on the viability and proliferation of HepG2 cells *in vitro*. The effect of plasma kallikrein was tested and compared between CCl_4_-treated and non–CCl_4_-treated cells. First, HepG2 cells treated with 1 or 2 mM of CCl_4_ for 24 h showed 40.8 and 60.5% cell death, respectively, whereas 2.5 ng/ml of plasma kallikrein showed no toxicity even at day 2 (Figures 2B,C). Likewise, plasma kallikrein increased the proliferation rate of HepG2 cells by 20.4 and 72.6% at day 1 and day 2 of treatment, respectively, (Figure 2C). In 1 mM CCl_4_-treated cells, plasma kallikrein elicited a 45.3% significant increase in proliferation compared to the 1 mM CCl_4_-treated cells alone (*p* < 0.0001, Figure 2D). Incubation of 2 mM CCl_4_-treated HepG2 cells with plasma kallikrein did not cause an increase in proliferation (Figure 2D), possibly due to the high toxicity of 2 mM CCl_4_ on the HepG2 cells. These results suggest a role in the proliferation of normal and necrosis-affected cells either through normally or necrosis-released molecular patterns. In parallel, we determined the effect of plasma kallikrein on HepG2 and observed a statistically significant induction of interleukin 1 beta (IL1B) and F2R genes but not PAR2 (F2RL1) gene expression (Figure 2D).

### The Induction of PKKS Genes Is Highly Associated to Inflammation and Immune Cell Recruiting Genes in the Liver

**FIGURE 3:**
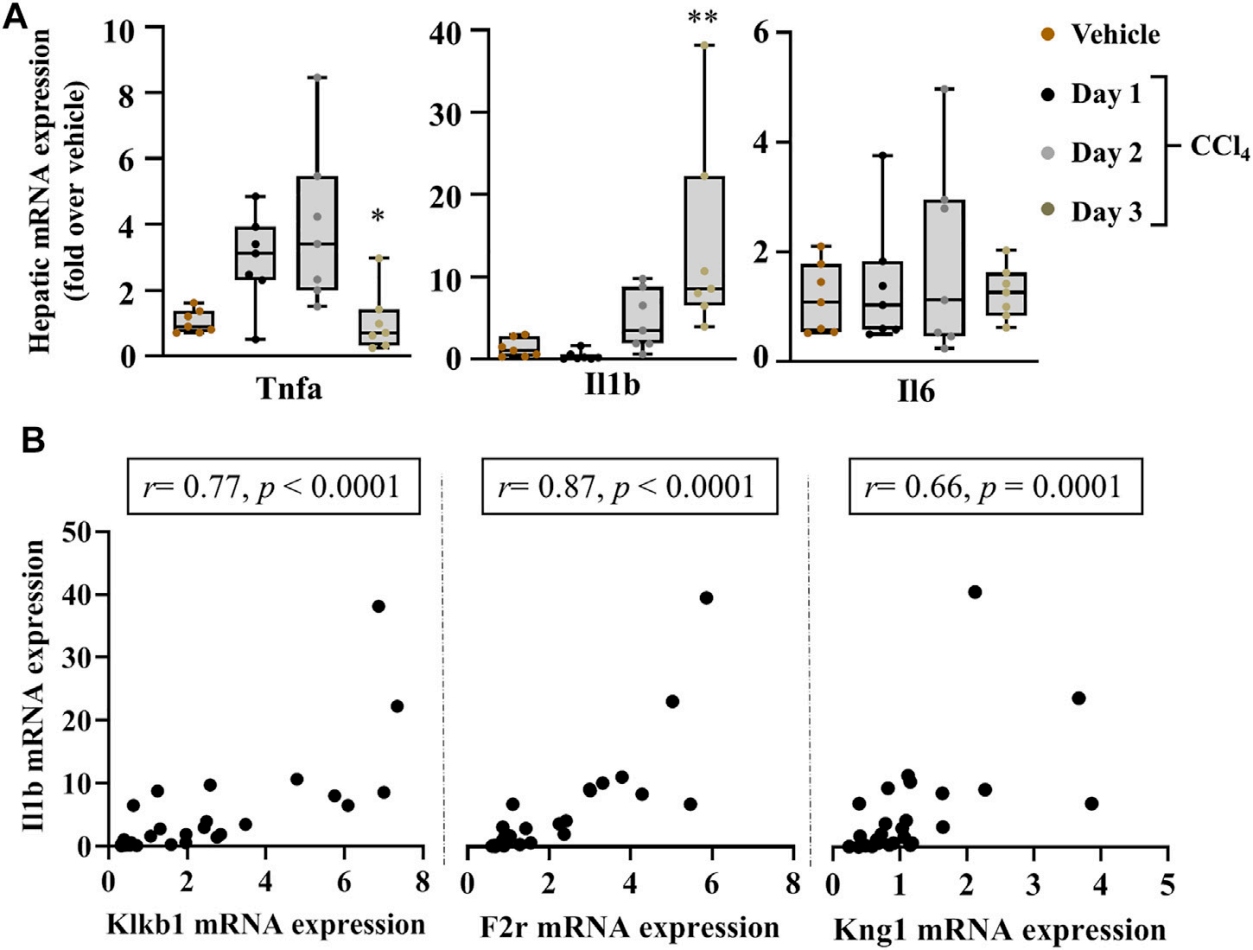
Strong association of PKKS genes to hepatic mRNA levels of inflammatory markers in acute liver injury. **(A)** Tnfa, Il1b, and Il6 gene induction implicates an inflammatory response to liver injury over the time course observed. **(B)** Spearman correlation studies between the Klklb1 gene and Ilb gene, the F2r gene and Il1b gene, and the Kng1 gene and Il1b gene. Data representation and statistical analysis were performed as described in the legend for Figure 1C (*n* = 7 per group). *p < 0.05, **p < 0.005 (CCl_4_ vs. Vehicle), and correlative plots were assessed by the Spearman correlation. Vehicle corresponds to mineral oil.

**FIGURE 4:**
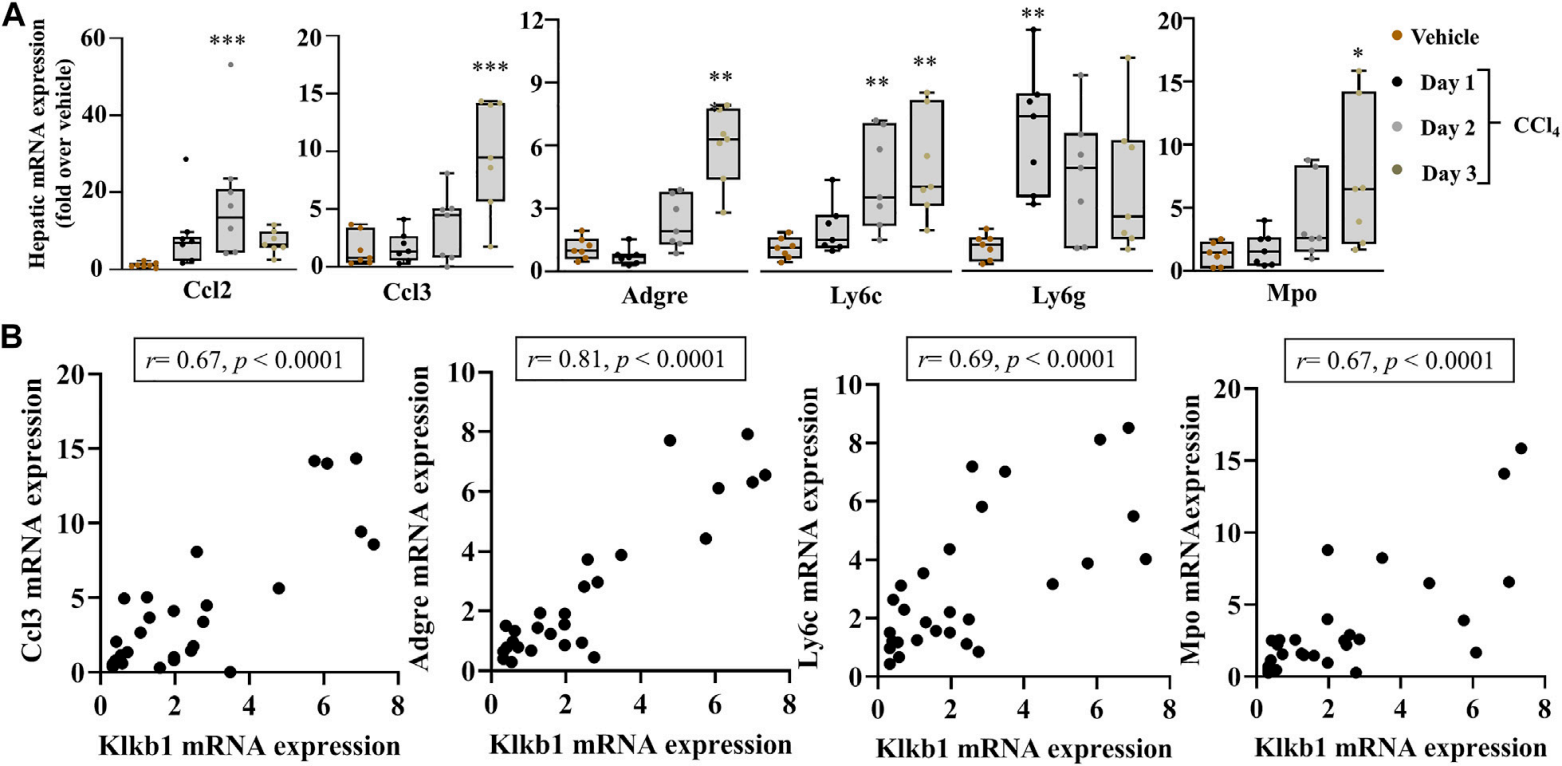
Strong association of immune cells recruiting markers in acute liver injury. **(A)** Chemokine genes, Ccl2 and Ccl3; recruitment genes Adgre1, Ly6c, and Ly6g; and Mpo gene, a marker of neutrophil activation. **(B)** The correlation analysis by Spearman among the Klkb1 and Ccl3 genes; the Klkb1 and Adgre1 genes; the Klkb1 and Ly6c genes; and the Klkb1 and Mpo genes. Data representation and statistical analysis were performed as described in the legend for Figure 1C. Outliers are shown by dots outside the whiskers (*n* = 7 per group). *p < 0.05, **p < 0.005, ***p < 0.0005 (CCl_4_ vs. Vehicle), and correlative plots were assessed by Spearman correlation. Vehicle corresponds to mineral oil.

The effectors of PKKS, plasma kallikrein, and bradykinin on their receptors have been implicated in vascular and tissue inflammation. Figure 3A shows an increase in the mRNA levels of the inflammatory players, tumor necrosis factor-alpha (Tnfa) and Interleukin 1b (Il1b) genes in the liver, starting at day 1 after CCl_4_ injection, and with a maximal expression at day 2 for Tnfa, and day 3 for Il1b. Interleukin-6 (Il16) gene was not induced at the observed time point of acute liver injury. Spearman correlation studies with the PKKS and thrombin genes (F2) indicate a strong relationship with the Il1b gene (Figure 3B, Supplementary Figures 1A,B). Moreover, the expression of chemokine (C-C motif 2 and 3) Ccl2 and Ccl3 genes that may trigger the recruitment of immune cells to the site of injury (Mossanen et al., 2016; Reichel et al., 2012) was analyzed. Figure 4A indicates an increase in Ccl2- and Ccl3-mRNA expressions, which peaked at day 2 for Ccl2 and day 3 for Ccl3. Ccl3 was strongly associated to the PKKS genes as observed by the correlation coefficient (*r* = 0.67, 0.57, and 0.77, for Klkb1, Kng1, and F2r, respectively) (Figure 4B, Supplementary Figures 1A,B). Different subsets of immune cells have been detected at the acute injury site. Genes for the adhesion G protein–coupled receptor E1 (Adgre1), a marker of macrophage, lymphocyte antigen 6 complex, locus C1 and G, Ly6c, and Ly6g (markers of monocyte and neutrophil presence) have been shown to increase their expression in acute liver injury and inflammation (Mitchell et al., 2009; Endig et al., 2019). We assessed the possibility of the PKKS gene association to these immune cell recruiting genes. The mRNA expression levels of Adgre1, Ly6c, and Ly6g were increased post CCl_4_ with a transient increase for the Ly6g and a sustained increase for Adgre1 and Ly6c (Figure 4A). The Adgre1 and Ly6c genes showed a strong association to the PKKS genes (Figure 4B, Supplementary Figures 1A,B). Although the Ly6g gene showed no association to the PKKS genes, (Supplementary Figures 1A,B), the expression of myeloperoxidase gene, Mpo, a marker of neutrophil activation, gradually increased (Figure 4A), and a strong positive correlation with the PKKS genes (Figure 4B, Supplementary Figures 1A,B) was observed.

### The Induction of PKKS Genes

Plasma kallikrein induces an upregulation of the gene for galectin-3 (lectin, galactose binding, soluble 3, and LGALS3) in HepG2 cells.

**FIGURE 5:**
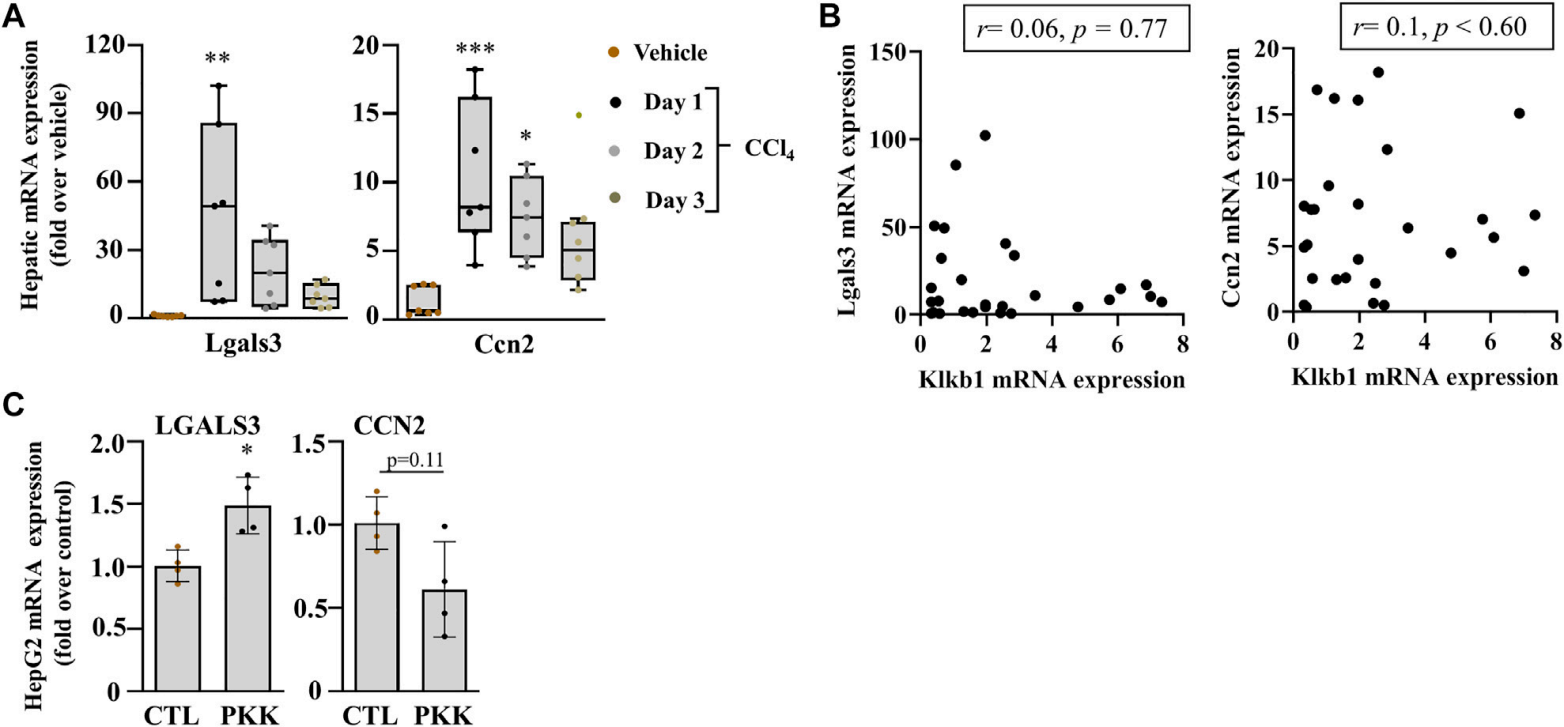
Early induction of myofibroblast markers of activation in acute liver injury. **(A)** mRNA expression of Lgals3 and Ccn2. Data representation and statistical analysis were performed as described in the legend for Figure 1C. (*n* = 7 per group). Outliers are shown by dots outside the whiskers, *p < 0.05**p < 0.005, ***p < 0.0005 (CCl_4_ vs. Vehicle). **(B)** Association of Klkb1, and Lgals3 and Ccn2, correlative plots were assessed by Spearman correlation. Vehicle corresponds to mineral oil. **(C)** Gene expression of LGALS3 and CCN2 in HepG2 cells were induced with plasma kallikrein (PKK). Data are shown as mean ± SEM (*n* = 4 per group), and statistical significance was determined by the Mann–Whitney test; *p < 0.05 (PKK vs. CTL). CTL corresponds to culture media only.

We analyzed the gene expression of myofibroblast activators, Lgals3 and Ccn2, the gene for connective tissue growyh factor (CTGF), and observed their increase in the mRNA level at day 1 after acute liver injury and a gradual decrease at day 2 till 3 in C57Bl/6J mice (Figure 5A). These genes showed no association with the PKKS genes (Figure 5B, Supplementary Figures 2A,B). Incubation of HepG2 cells with plasma kallikrein increased LGALS3 and decreased CCN2 gene expressions (Figure 5C), suggesting the involvement of plasma kallikrein in the modulation of these two myofibroblast activators in fibrosis.

### Fibrogenesis and Inflammation in Early Chronic Liver Injury: Regulation of Induced PKKS Genes

**FIGURE 6:**
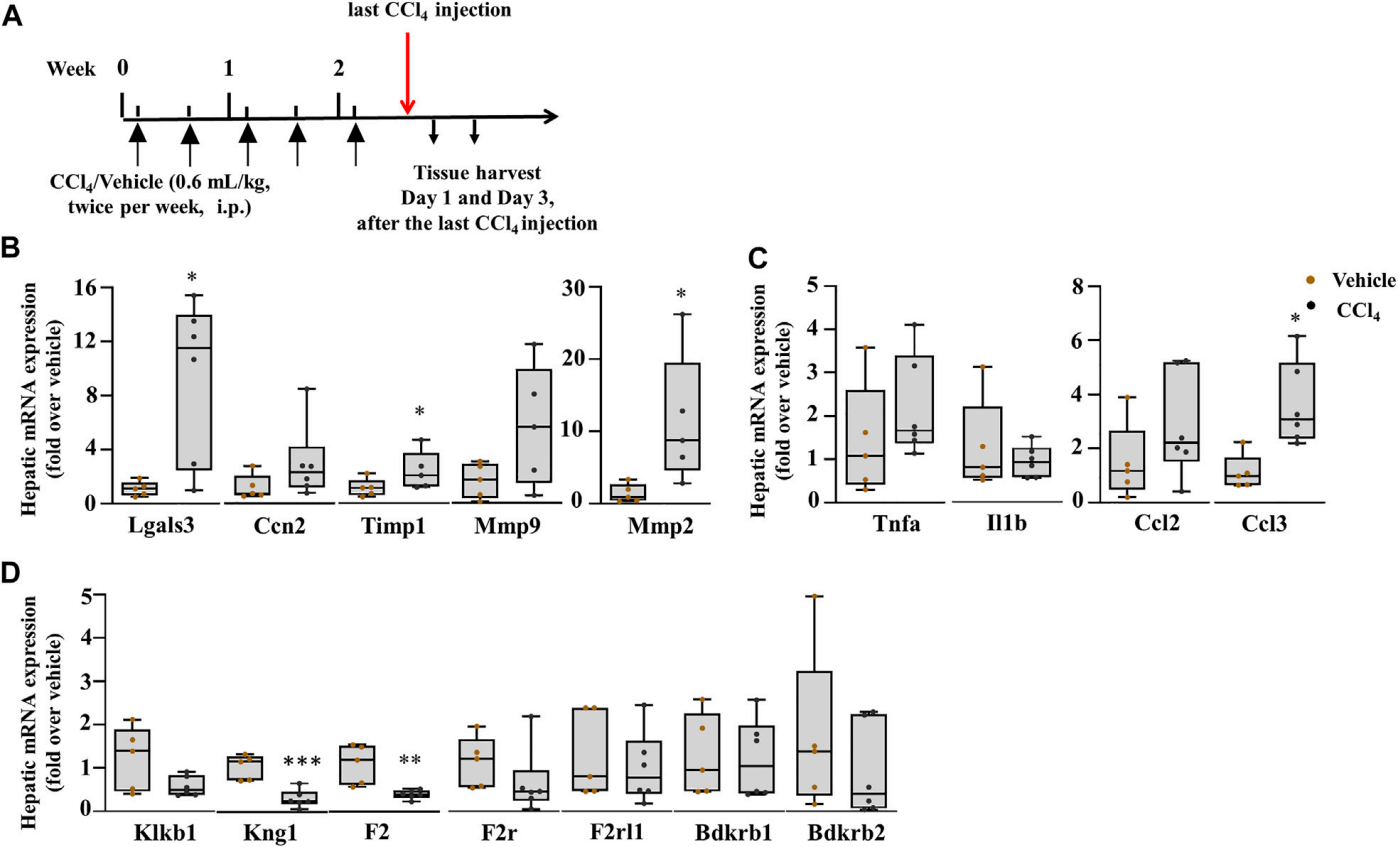
Upregulation of markers of fibrosis: inflammatory processes are regulated. **(A)** Schematic representation of CCl_4_-induced chronic liver injury. Hepatic mRNA expression of **(B)** Lgals3, Ccn2, Timp1, Mmp9, and Mmp2; **(C)** inflammatory and chemokine genes, Tnfa, Il1b, Ccl2, and Ccl3; **(D)** the PKKS genes and thrombin (F2). Data representation for box plots was performed as described in the legend for Figure 1C (*n* = 5–6 mice per group); statistical significance was determined by the Mann–Whitney test, where *p < 0.05, **p < 0.005 (CCl_4_ vs. Vehicle). Vehicle corresponds to mineral oil.

To investigate the role of plasma kallikrein in chronic liver injury, liver fibrosis was induced in C57BL/6J mice after subjection to 0.6 ml/kg of CCl_4_ for 2.5 weeks (Figure 6A). Exposure of the mice to CCl_4_ showed an increase in picro sirius red staining (Supplementary Figure 3A). In this setting, we analyzed the gene expression of hepatic Lgals3, Ccn2, and extracellular matrix remodeling players. Figure 6B showed a statistically significant increase in the mRNA expression of Lgals3, tissue inhibitor of metalloproteinase 1 (Timp1), and matrix metallopeptidase 2 (Mmp2) genes. Hepatic injury was assessed after the last CCl_4_ injection by H&E staining (Supplementary Figure 2B, left; delineated area) and showed a 29.4% increase in the necrotic area when compared to vehicle (Supplementary Figure 2B). The mRNA level of Hmgb1 was unchanged in this time point of liver fibrosis (Supplementary Figure 2C). Assessment of chemokine genes showed a statistically significant increase for the Ccl3 gene (Figure 6C). Ccl2, Tnfa, and Il1b remained unchanged (Figure 6C). Likewise, the Mpo gene revealed an unchanged gene expression compared to vehicle-treated animals (Supplementary Figure 2C). These results suggest the regulation of some inflammatory processes and fibrogenesis, contributing to ECM deposition. This is also observed in the PKKS genes where some genes were significantly downregulated (Kng1 and F2), and others with a tendency to decrease (Klkb1 and F2r) and unchanged (F2rl1, Bdkrb1, and Bdkrb2) (Figure 6D).

### Correlation Analysis of Klkb1 and Kng1 Gene Expression Suggests Different Roles at Different Time Points of Liver Fibrosis

**FIGURE 7:**
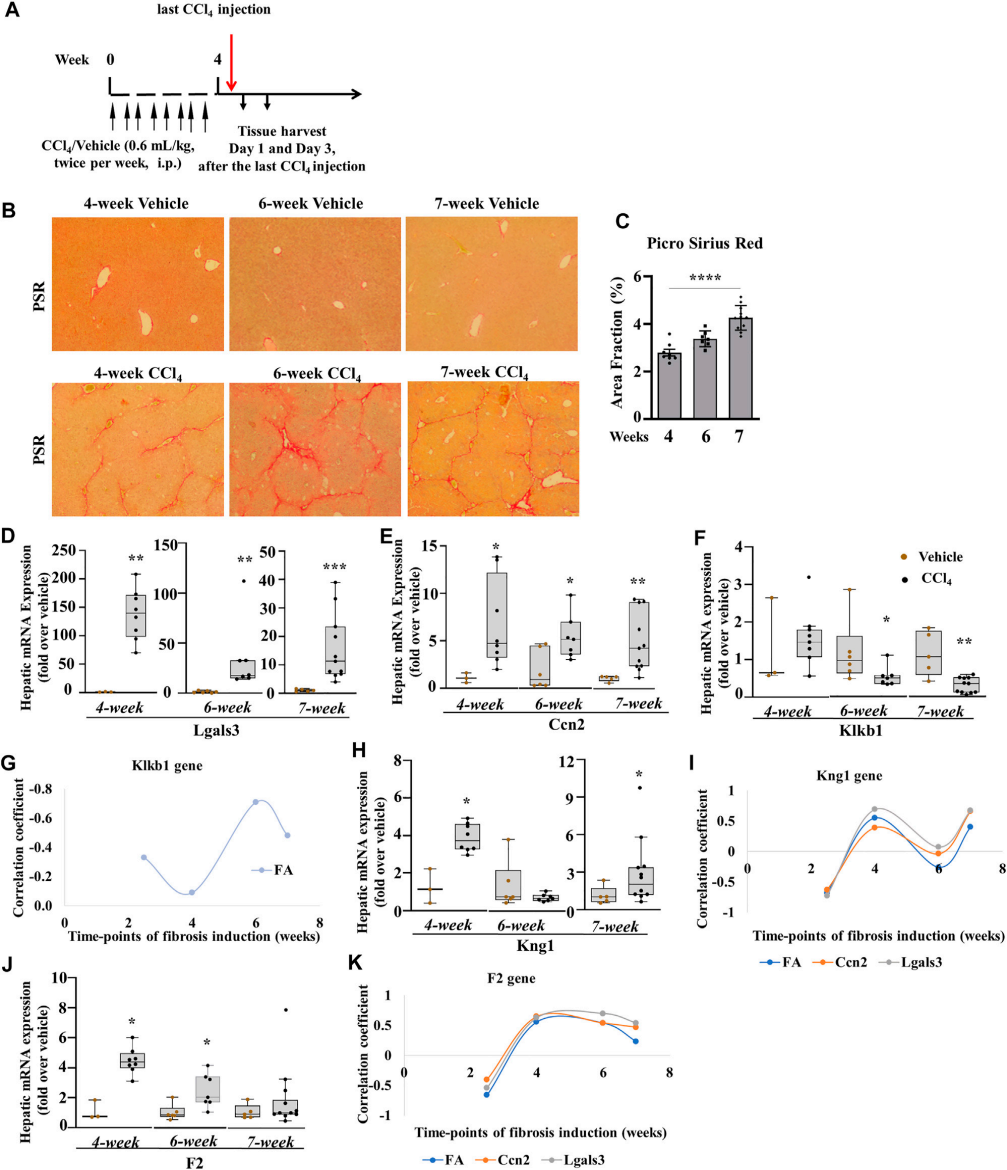
Klkb1 and Kng1 gene expressions at different time points of the chronic liver injury model. **(A)** Schematic representation of CCl_4_-induced liver fibrosis at the 4th week. Similar pattern of mice injections was performed at the 6th and 7th weeks as well. **(B)** Varying representative images of collagen fibers stained with picro sirius red (stained red). **(C)** Quantification of histological staining by ImageJ software (data representation by mean ± SEM, *n* = 3–10 mice per group: one-way ANOVA followed by Sidak’s multiple comparisons; ****p < 0.0001). The quantified areas in each sample were normalized to their average time points of controls before statistical analysis. Hepatic mRNA expression **(D, E, F)** of Lgals3, Ccn2, and Klkb1; **(G)** plot analysis of Klkb1 gene and **(H)** Kng1 gene; **(I)** plot analysis of Kng1 gene and **(J)** F2 gene; **(K)** plot analysis of F2 gene. Data representation for box plots was performed as described in the legend for Figure 1C. Outliers are shown by dots outside the whiskers (*n* = 3–6 mice for Vehicle and 7–10 for CCl_4_ treatment groups); statistical significance was determined by the Mann–Whitney test, where *p < 0.05, **p < 0.005, ***p < 0.0005, ****p < 0.0001 (CCl_4_ vs. Vehicle). Vehicle corresponds to mineral oil.

To corroborate the results of liver fibrogenesis on the PKKS effectors (plasma kallikrein and KNG1) and thrombin in chronic liver injury, we studied different time points of CCl_4_-induced liver fibrosis (4, 6, and 7 weeks). We analyzed their association to the myofibroblast activation markers, and fibrotic areas at all time points of chronic liver injury, including 2.5 weeks. The mice were treated with the same volume of vehicle or CCl_4_ twice per week and sacrificed after the last injection (Figure 7A; similar treatment pattern was performed at the 6th and 7th weeks). Fibrosis was established at all time points (Figure 7B). There was a gradual increase in sirius red staining from approximately 3.0 folds in the 4-week time point to 3.4 and 4.2 folds in the 6- and 7-week time points, respectively (Figure 7C). The genes of myofibroblast activation markers, Lgals3 and Ccn2, were increased at all time points (Figures 7D,E). Klkb1 gene decreased at 6- and 7-week time points (Figure 7F). Supplementary Figures 3A–D showed an insignificant weak relationship between the Klkb1 mRNA expression to fibrotic area or gene expressions of Lgals3 and Ccn2 across all time points, except for the fibrotic area at 6 weeks (*r* = 0.71, *p* = 0.05, Figure 7G). We hypothesized a similar pattern of gene expression of Kng1 and Klkb1 mRNA levels due to their complex formation in the plasma and stoichiometric interaction (Kusumam et al., 2009; Kaplan and Ghebrehiwet, 2010). However, the Kng1 gene (Figure 7H) displayed a significant upregulation across all CCl_4_ time points except at the 6-week time point. Correlation analysis of the Kng1 gene showed a strong relationship at all time points, except at the 6-week time point, suggesting a more involved and different role compared to the Klkb1 gene (Figure 7I, Supplementary Figures 3A–D, 4A–D). We showed that plasma kallikrein modulates Ccn2 gene expression (Figure 5C) and differs from thrombin in acute liver injury. In the observed time points, the gene expression of thrombin (F2) gene (Figure 7J) increased at the 4th and 6th weeks and formed a more robust correlative relationship compared to the Klkb1 gene expression (Figure 7K, Supplementary Figures 3A–D, 4A–D).

### The PKKS Receptors: A Potential Role of Bradykinin Receptors in Liver Fibrosis

**FIGURE 8:**
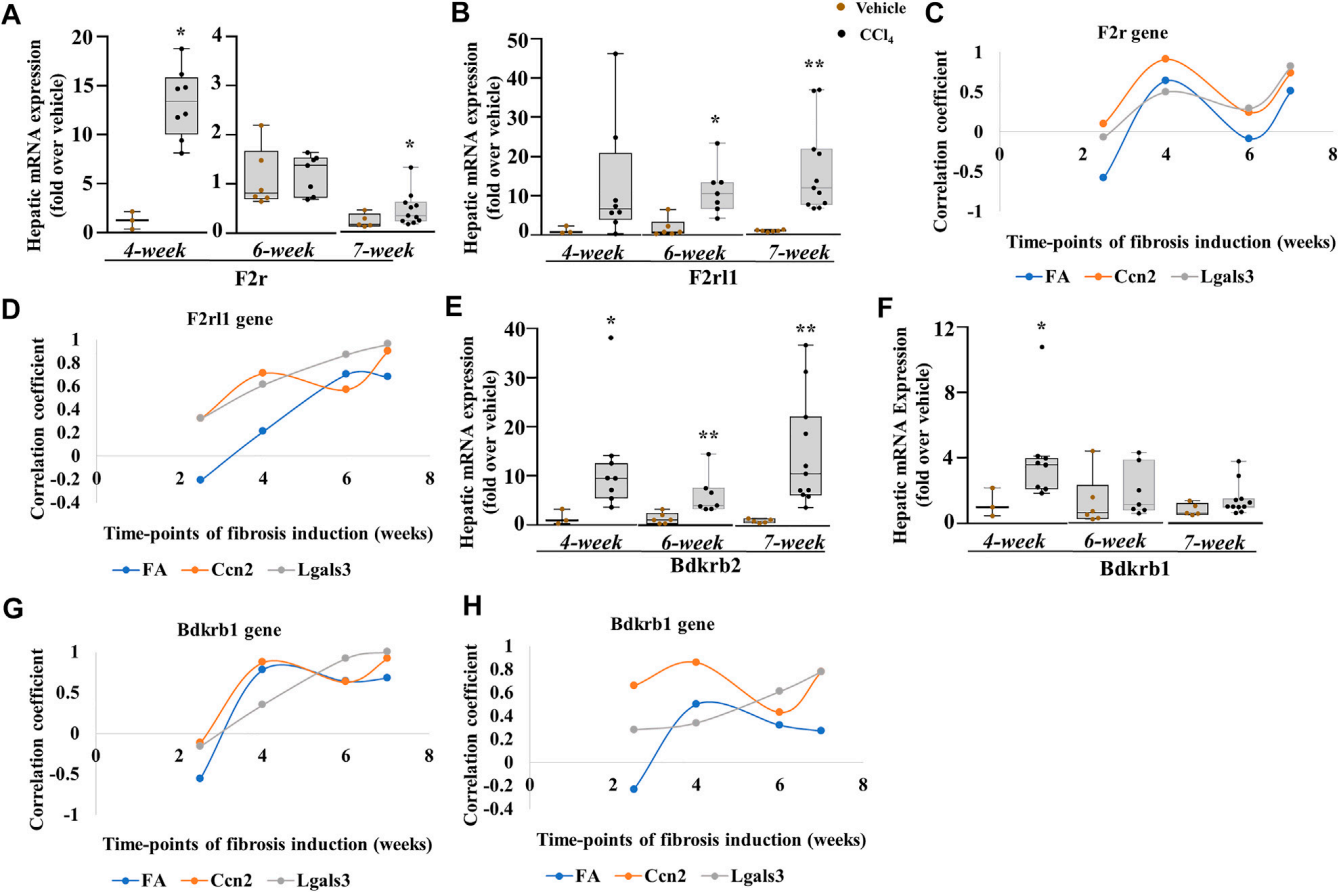
PKKS receptors at different time points of the chronic injury model. **(A, B)** F2r and F2rl1 genes. **(C, D)** Plot analysis of the association among F2r, F2rl1, and Lgals3, Ccn2 genes, and fibrotic area (FA). Hepatic mRNA expression of **(E, F)** Bdkrb2 and Bdkrb1. **(G, H)** Plot analysis of the association among Bdkrb2, Bdkrb1, and Lgals3, Ccn2 genes, and fibrotic area (FA). Data representation for box plots was performed as described in the legend for Figure 1C. Outliers are shown by dots outside the whiskers (*n* = 3–6 mice for Vehicle and 7–10 for CCl_4_ treatment groups); statistical significance was determined by the Mann–Whitney test, where *p < 0.05; **p < 0.005; ***p < 0.0005 (CCl_4_ vs. Vehicle) Vehicle corresponds to mineral oil.

Finally, we assessed the receptors of the PKKS. F2r mRNA expression (Figure 8A) increased significantly at the early and progressive stages of liver fibrosis, whereas the F2rl1 mRNA level (Figure 8B) that was unchanged in acute and after 2.5-week liver injury increased at the 4th, 6th, and 7th weeks of CCl_4_ treatment, suggesting a delayed induction. The F2r gene (Figure 8C, Supplementary Figures 3A–D, 4A–D) exhibited more relationship at the 4th and 7th weeks, while the F2rl1 gene (Figure 8D, Supplementary Figures 3A–D, 4A–D) showed positive correlation with the Lgals3 and Ccn2 genes, and the fibrotic area across 4- and 7-week time points. Compared to the Klkb1 gene, the F2r and F2rl1 genes were strongly associated to the myofibroblast activators and fibrotic areas, thus confirming the involvement of other ligands. An increase in the Bdkrb2 gene expression over all time points was observed while the Bdkrb1 gene expression peaked at the 4th week (Figures 8E,F). Analysis by correlation showed a strong relationship of the Bdrkb2 gene with the myofibroblast activation markers and the fibrotic area in the early and progressive stages of liver fibrosis, thereby strengthening the relationship of the Kng1 gene to liver fibrosis and conferring a possible similar pattern of regulation of the Bdkrb2 and Kng1 genes (Figure 8G; Supplementary Figures 3A–D, 4A–D).

## Discussion

Many studies have investigated mechanisms driving hepatic injury and fibrosis, extracellular matrix remodeling, and regeneration pattern. These processes implicated different patterns of coagulation, cell death, inflammation, and myofibroblast activation. Yet, the connection among these patterns is incomplete as more are being implicated in the pathogenesis of liver injury and fibrosis. In this study, we examined the gene expression profile of the components of the PKKS in liver injury and fibrosis and associated their expression levels to pathways that influence liver disease such as cell death, inflammation, and fibrosis.

One important finding of this study is the significant expression of Klkb1, Kng1, and F2r mRNA levels in acute liver injury, which was positively associated with the increase in cell death marker, Hmgb1 gene expression, as well as pro-inflammatory and immune-recruiting markers such as Il1b, Ccl3, Adgre1, Ly6c, and neutrophil activation gene expression, Mpo. In addition, our data provided the first observation of the proliferation of hepatic HepG2 cells in response to plasma kallikrein, and the concomitant induction of inflammatory cytokine, IL1B, and myofibroblast activator genes CCN2 and LGALS3 in HepG2 cells, respectively. Last, the gene expression of Bdkrb2 was significantly induced in response to chronic liver injury in C57BL6/J mice and was positively correlated with the progression of liver fibrosis.

Few studies have addressed the role of PKKS in liver injury. Earlier studies by Borges et al. showed the increased clearance rate of plasma kallikrein by the exsanguinated liver in the murine model of acute inflammation (Martins et al., 1992), while it decreased in chronic liver injury (Toledo et al., 1995). Further investigation implicated a galectin-mediated pathway in endocytic clearance of the plasma kallikrein protein in the liver (Nagaoka et al., 2003). In this regard, our study showed that plasma kallikrein treatment stimulated the LGALS3 gene expression in hepatocytes. Intra- and extracellular galectin-3 play a role in the regulation of phagocytosis-induced activation of hepatic stellate cells (Jiang et al., 2012). Our study points to the upregulation of the mRNA expression of Lgals-3 at day 1 of the acute liver injury model and chronic liver injury, and suggests a potential role in liver injury. Furthermore, the temporal increase in the gene expressions of Klkb1 and F2r in acute liver injury may suggest a potential functional link between them. In addition, our data showed that plasma kallikrein treatment induced the gene expression of F2r and Il1b, and a concomitant increase in the proliferation of both untreated and 1 mM CCl_4_-treated HepG2 cells. The inability of plasma kallikrein to induce an increase in the proliferation of 2 mM CCl_4_-treated HepG2 cells could be attributed to the decreased viability limiting the proliferation of these cells. Other studies have shown that plasma kallikrein can promote the proliferation in synovial cells (Dai et al., 2012). In this regard, our data pointed to a strong association between the Klkb1 and Hmgb1 gene expressions. Chen et al. (2014) showed that HMGB1 acts as a driver of acute liver injury (Chen et al., 2014) and inflammation. The inflammatory roles of HMGB1 (El Gazzar, 2007) could potentially modulate the expressions of the PKKS as indicated by their positive association.

Several studies have implicated the infiltration of immune cells as drivers of liver injury (Weiskirchen et al., 2018; Dong, 2019; Zhangdi et al., 2019). The PKKS is involved in the recruitment of immune cells (Gobel et al., 2019), and our study revealed their positive association to gene expression of recruiting immune cells. The relationship between the PKKS and neutrophils as indicated by the Ly6g and Mpo gene expressions depicts an activated phenotype which could be involved in acute liver injury progression or resolution. The PKKS especially through plasma kallikrein and bradykinin contributes to neutrophil activation and thereby inflammation (Kenne et al., 2019). This activation and inflammation as previously described require the liver sinusoidal endothelial cells in propagating or resolving liver injury or fibrosis (Kenne et al., 2019; McDonald and Kubes, 2012). Interestingly, the clustering of plasma kallikrein around injured areas as seen by immunohistochemistry could be attributed to its release from immune cells, eventually promoting hepatocyte proliferation. Our result is the first to describe the cluster formation of plasma kallikrein around necrotic areas and the possible proliferation of hepatocytes by plasma kallikrein.

In our chronic liver injury model, which mimics liver fibrosis time points of fibrogenesis and fibrosis, a different pattern of the PKKS gene expression was observed compared to that of acute liver injury. The Klkb1 mRNA level showed a decreased expression over all time points with no association to the myofibroblast activators and collagen deposition. Yet, in liver inflammation and fibrosis, plasma kallikrein cleaves the transforming growth factor, beta 1 (Kuniharu et al., 2002; Hara et al., 2014). This observation and our study suggest a feedback regulation over the Klkb1 gene. Nevertheless, receptors for plasma kallikrein, F2r, and F2rl1, mRNA expression, were increased at all time points, except at 2.5 weeks. This was expected as they are upregulated along with agonists, thrombin, factor Xa, and mast tryptase (Borensztajn et al., 2008; Borensztajn et al., 2010; Knight et al., 2012; Nault et al., 2016; Rullier et al., 2008). PAR1 induces the upregulation of CCN2 (Rullier et al., 2008). Our study highlights that the regulation of F2r could have affected in part the Ccn2 gene expression. Also, while F2r gene expression showed a strong positive association at 4 and 7 weeks of fibrosis establishment, the F2rl1 gene expression delayed fibrosis to 4 weeks through 7 weeks. Since both genes are significantly associated to the myofibroblast activator, CCN2, this supports previous experiments of PAR1 as an inducer of CCN2. Although the total knockout of F2r gene did not completely abrogate the expression of the Ccn2 gene (Rullier et al., 2008), compensatory PAR-like players such as PAR3 and PAR4 may be involved. Also, it is established that a cross talk exists between PAR1 and PAR2, especially with the transforming growth factor, beta 1 system (Ungefroren et al., 2018). Here we described that the F2rl1 mRNA expression showed more association to fibrosis, especially at 6- and 7-week time points. This is also prominent with the Lgals3 mRNA expression at 4, 6, and 7 weeks. Our study reveals a potential relationship between F2rl1 and myofibroblast activators, Lgals3 and Ccn2, and the fibrotic area in early, progressive, and late fibrosis.

Furthermore, the Kng1 gene expression showed a strong negative association to mRNA levels of Lgals3, Ccn2, and fibrotic area, except at the 6-week time point, corresponding to the relationship observed in acute liver injury. Kng1 gene expression is likely to be linked to liver fibrosis as supported by studies showing the inhibition of thrombin-induced platelet aggregation by bradykinin in humans (Murphey et al., 2006). Likewise, Sancho-Bru et al. (2007) showed that the infusion of bradykinin in extracted rat hepatocytes corrected hepatocellular damage in a chronic liver injury model (Sancho-Bru et al., 2007). However, in another experimental model of trichloroethylene induction of liver injury, bradykinin activated the Kupffer cells through BDKRB2 and contributed to liver injury (Zhang et al., 2019). This shows the varying interactions of the PKKS with various cell types within the liver architecture. However, our data showed that bradykinin receptors, especially Bdkrb2 mRNA level, are positively associated with liver fibrosis, implicating a potential role for these receptors in liver injury.

**FIGURE 9:**
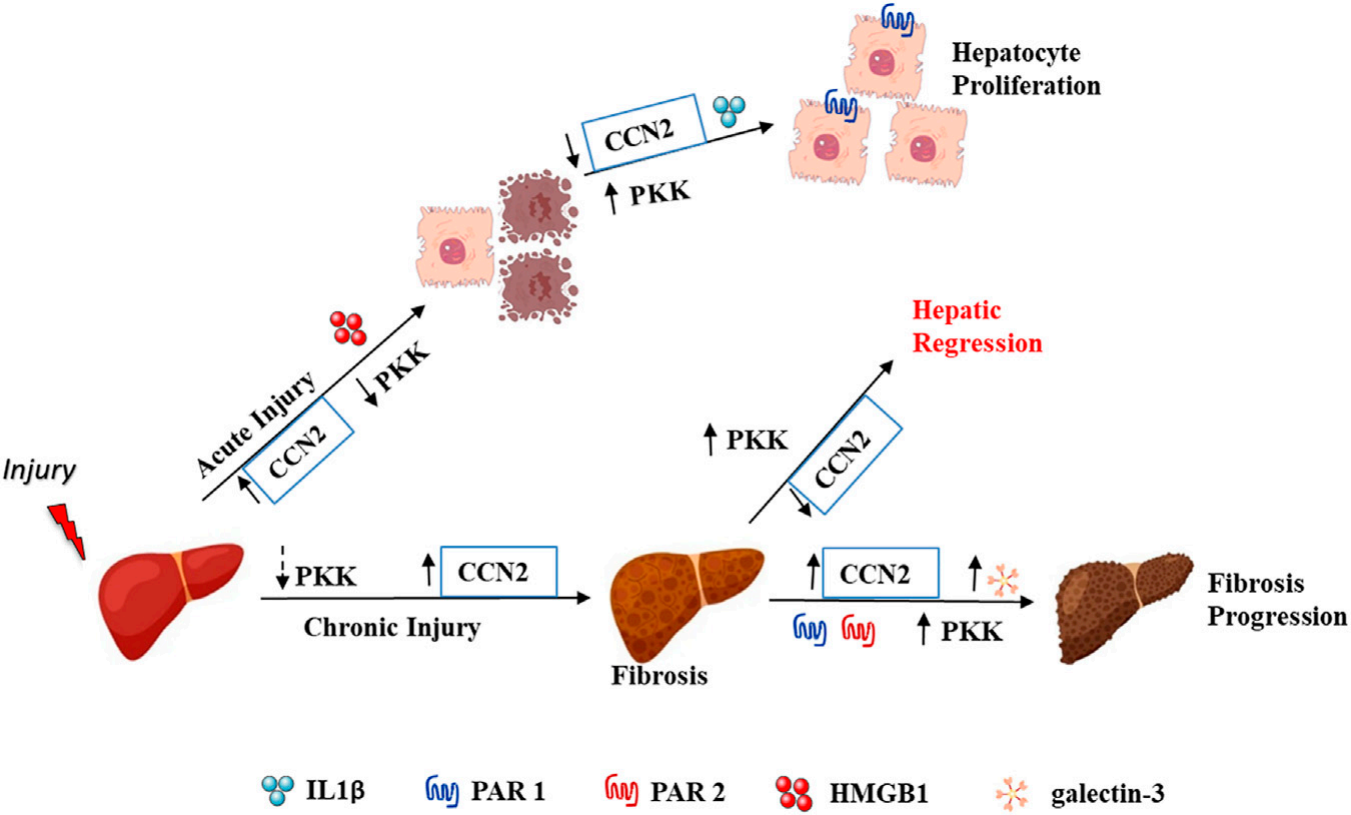
Plasma kallikrein-kinin system diverse roles in liver injury.

Overall, our findings implicated the involvement of plasma kallikrein in the hepatic milieu by stimulating LGALS3, CCN2, and IL1B, and suggest a regulation of the increase in proliferation of hepatocytes—a factor needed in repopulating hepatocytes in liver regeneration (Figure 9). Although these findings were done on cultured cells, our *in vivo* study showed the presence of plasma kallikrein in clusters around necrotic areas of the injured liver. This indicates its possible role in either protective or wound resolution processes. We are currently conducting studies exploring the function of plasma kallikrein in the liver, in terms of cause and effect, knowing fully well that an injured liver impairs many of its hepatic roles. Our future investigation is to relate plasma kallikrein to important functions of the liver such as metabolism, detoxification, inflammation, immunity, and blood coagulation. This will involve pharmacological inhibition or invalidation of the plasma kallikrein gene. In the present study, we also identified new relationships and interactions among the following: Kng1, F2r, F2rl1, Bdkrb1, and Bdkrb2 mRNA expression to Ccn2 and Lgals3 mRNA levels, and fibrosis in relation to chronic liver injury. This development among Kng1, Bdkrb1, and Bdkrb2 gene expressions, and myofibroblast activators and chronic liver injury creates a new direction in the study of liver fibrosis and its resolution. It is important to define the functional role of these genes in the development and progression of liver injury in order to identify new targets for intervention.

## Data Availability

The original contributions presented in the study are included in the article/Supplementary Material; further inquiries can be directed to the corresponding author.
