# Supplementary material for: Plasma Kallikrein as a Modulator of Liver Injury/Remodeling
Source: Front Pharmacol. 2021 Sep 9;12:715111. doi: 10.3389/fphar.2021.715111 (PMC8458624; doi:10.3389/fphar.2021.715111)
Supplement: Supplementary file 1 [file Presentation1.PPTX]

## Slide 1
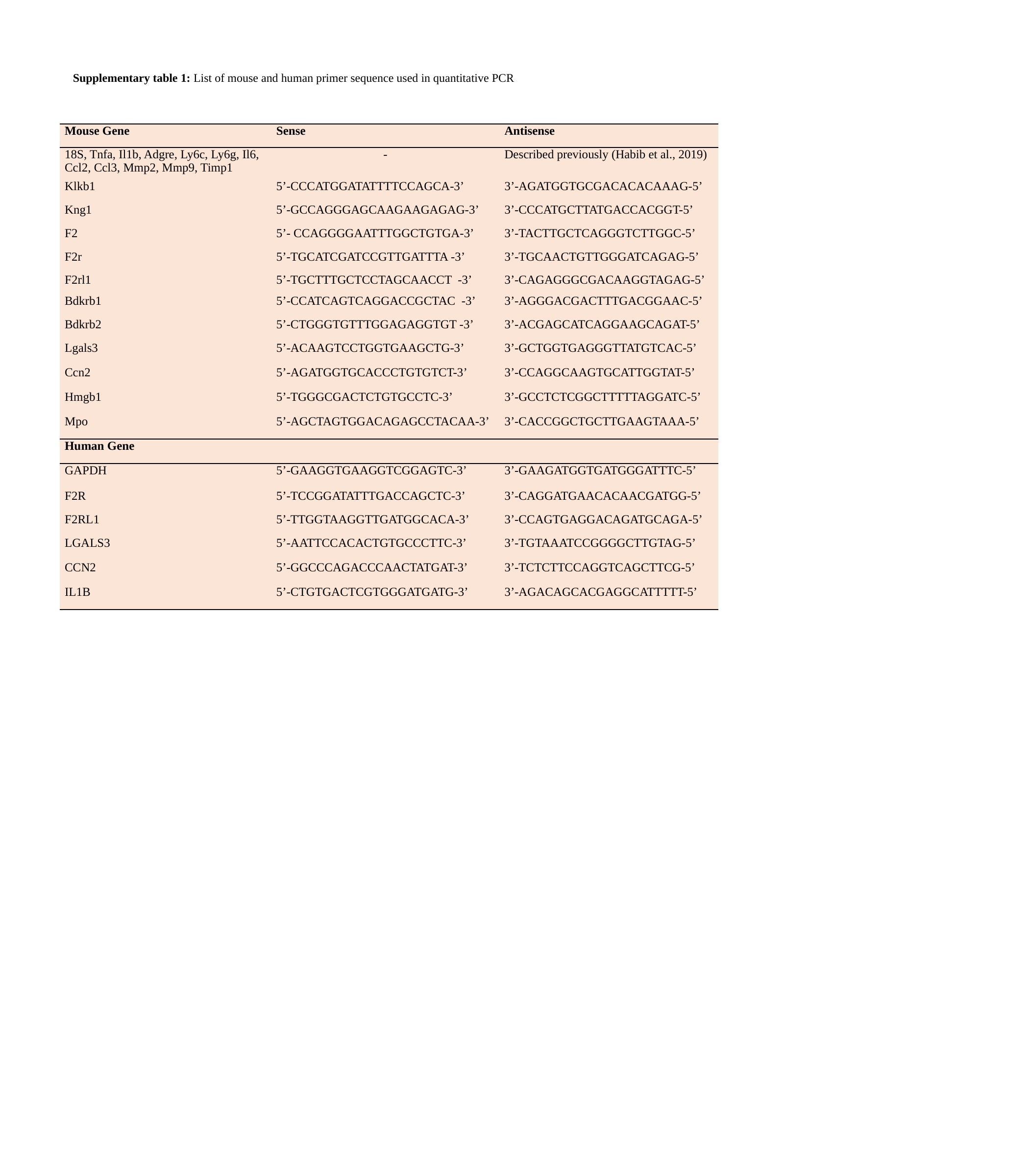

Supplementary table 1: List of mouse and human primer sequence used in quantitative PCR
| Mouse Gene | Sense | Antisense |
| --- | --- | --- |
| 18S, Tnfa, Il1b, Adgre, Ly6c, Ly6g, Il6, Ccl2, Ccl3, Mmp2, Mmp9, Timp1 | - | Described previously (Habib et al., 2019) |
| Klkb1 | 5’-CCCATGGATATTTTCCAGCA-3’ | 3’-AGATGGTGCGACACACAAAG-5’ |
| Kng1 | 5’-GCCAGGGAGCAAGAAGAGAG-3’ | 3’-CCCATGCTTATGACCACGGT-5’ |
| F2 | 5’- CCAGGGGAATTTGGCTGTGA-3’ | 3’-TACTTGCTCAGGGTCTTGGC-5’ |
| F2r | 5’-TGCATCGATCCGTTGATTTA -3’ | 3’-TGCAACTGTTGGGATCAGAG-5’ |
| F2rl1 | 5’-TGCTTTGCTCCTAGCAACCT -3’ | 3’-CAGAGGGCGACAAGGTAGAG-5’ |
| Bdkrb1 | 5’-CCATCAGTCAGGACCGCTAC -3’ | 3’-AGGGACGACTTTGACGGAAC-5’ |
| Bdkrb2 | 5’-CTGGGTGTTTGGAGAGGTGT -3’ | 3’-ACGAGCATCAGGAAGCAGAT-5’ |
| Lgals3 | 5’-ACAAGTCCTGGTGAAGCTG-3’ | 3’-GCTGGTGAGGGTTATGTCAC-5’ |
| Ccn2 | 5’-AGATGGTGCACCCTGTGTCT-3’ | 3’-CCAGGCAAGTGCATTGGTAT-5’ |
| Hmgb1 | 5’-TGGGCGACTCTGTGCCTC-3’ | 3’-GCCTCTCGGCTTTTTAGGATC-5’ |
| Mpo | 5’-AGCTAGTGGACAGAGCCTACAA-3’ | 3’-CACCGGCTGCTTGAAGTAAA-5’ |
| Human Gene | | |
| GAPDH | 5’-GAAGGTGAAGGTCGGAGTC-3’ | 3’-GAAGATGGTGATGGGATTTC-5’ |
| F2R | 5’-TCCGGATATTTGACCAGCTC-3’ | 3’-CAGGATGAACACAACGATGG-5’ |
| F2RL1 | 5’-TTGGTAAGGTTGATGGCACA-3’ | 3’-CCAGTGAGGACAGATGCAGA-5’ |
| LGALS3 | 5’-AATTCCACACTGTGCCCTTC-3’ | 3’-TGTAAATCCGGGGCTTGTAG-5’ |
| CCN2 | 5’-GGCCCAGACCCAACTATGAT-3’ | 3’-TCTCTTCCAGGTCAGCTTCG-5’ |
| IL1B | 5’-CTGTGACTCGTGGGATGATG-3’ | 3’-AGACAGCACGAGGCATTTTT-5’ |

## Slide 2
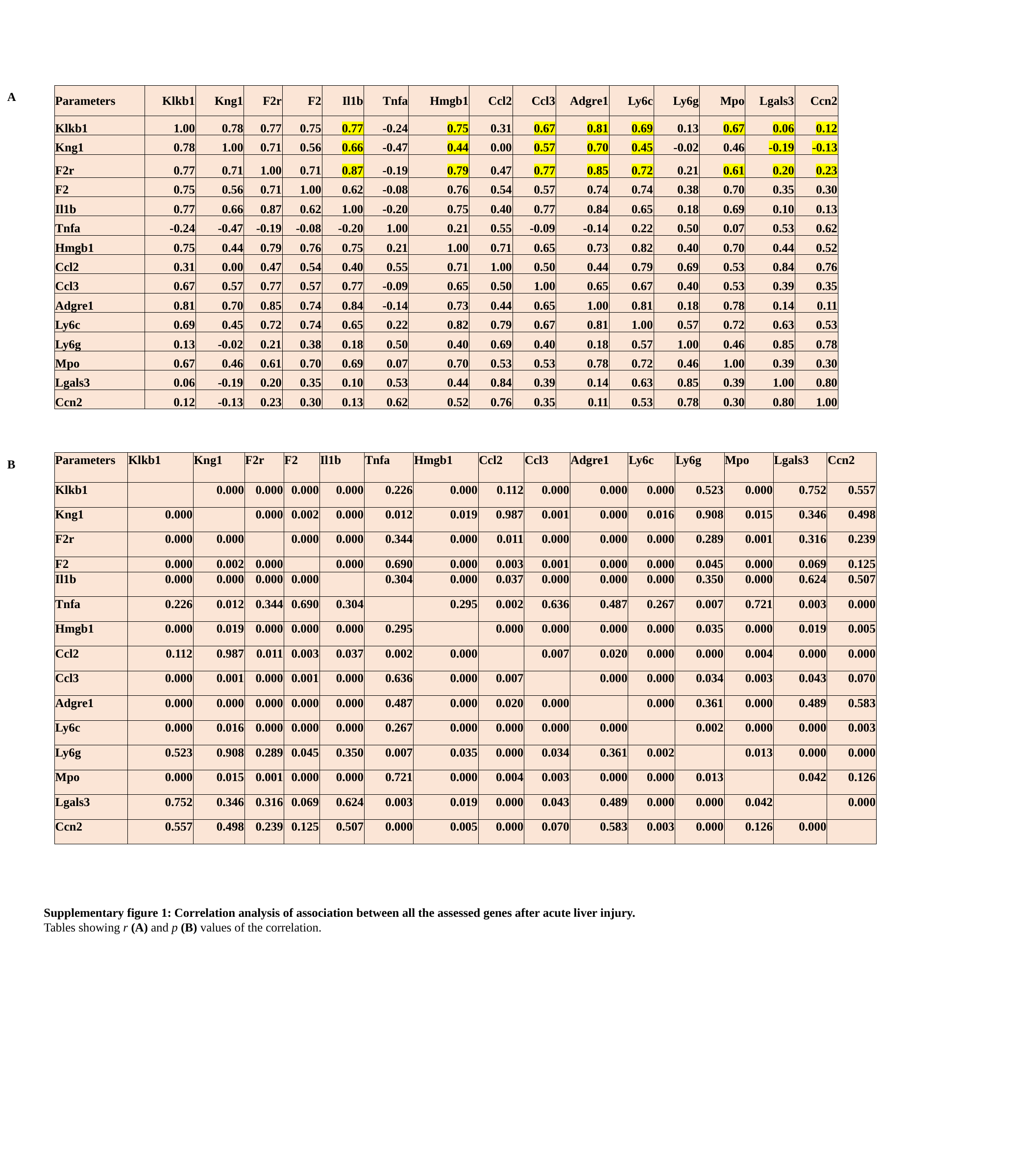

A
| Parameters | Klkb1 | Kng1 | F2r | F2 | Il1b | Tnfa | Hmgb1 | Ccl2 | Ccl3 | Adgre1 | Ly6c | Ly6g | Mpo | Lgals3 | Ccn2 |
| --- | --- | --- | --- | --- | --- | --- | --- | --- | --- | --- | --- | --- | --- | --- | --- |
| Klkb1 | 1.00 | 0.78 | 0.77 | 0.75 | 0.77 | -0.24 | 0.75 | 0.31 | 0.67 | 0.81 | 0.69 | 0.13 | 0.67 | 0.06 | 0.12 |
| Kng1 | 0.78 | 1.00 | 0.71 | 0.56 | 0.66 | -0.47 | 0.44 | 0.00 | 0.57 | 0.70 | 0.45 | -0.02 | 0.46 | -0.19 | -0.13 |
| F2r | 0.77 | 0.71 | 1.00 | 0.71 | 0.87 | -0.19 | 0.79 | 0.47 | 0.77 | 0.85 | 0.72 | 0.21 | 0.61 | 0.20 | 0.23 |
| F2 | 0.75 | 0.56 | 0.71 | 1.00 | 0.62 | -0.08 | 0.76 | 0.54 | 0.57 | 0.74 | 0.74 | 0.38 | 0.70 | 0.35 | 0.30 |
| Il1b | 0.77 | 0.66 | 0.87 | 0.62 | 1.00 | -0.20 | 0.75 | 0.40 | 0.77 | 0.84 | 0.65 | 0.18 | 0.69 | 0.10 | 0.13 |
| Tnfa | -0.24 | -0.47 | -0.19 | -0.08 | -0.20 | 1.00 | 0.21 | 0.55 | -0.09 | -0.14 | 0.22 | 0.50 | 0.07 | 0.53 | 0.62 |
| Hmgb1 | 0.75 | 0.44 | 0.79 | 0.76 | 0.75 | 0.21 | 1.00 | 0.71 | 0.65 | 0.73 | 0.82 | 0.40 | 0.70 | 0.44 | 0.52 |
| Ccl2 | 0.31 | 0.00 | 0.47 | 0.54 | 0.40 | 0.55 | 0.71 | 1.00 | 0.50 | 0.44 | 0.79 | 0.69 | 0.53 | 0.84 | 0.76 |
| Ccl3 | 0.67 | 0.57 | 0.77 | 0.57 | 0.77 | -0.09 | 0.65 | 0.50 | 1.00 | 0.65 | 0.67 | 0.40 | 0.53 | 0.39 | 0.35 |
| Adgre1 | 0.81 | 0.70 | 0.85 | 0.74 | 0.84 | -0.14 | 0.73 | 0.44 | 0.65 | 1.00 | 0.81 | 0.18 | 0.78 | 0.14 | 0.11 |
| Ly6c | 0.69 | 0.45 | 0.72 | 0.74 | 0.65 | 0.22 | 0.82 | 0.79 | 0.67 | 0.81 | 1.00 | 0.57 | 0.72 | 0.63 | 0.53 |
| Ly6g | 0.13 | -0.02 | 0.21 | 0.38 | 0.18 | 0.50 | 0.40 | 0.69 | 0.40 | 0.18 | 0.57 | 1.00 | 0.46 | 0.85 | 0.78 |
| Mpo | 0.67 | 0.46 | 0.61 | 0.70 | 0.69 | 0.07 | 0.70 | 0.53 | 0.53 | 0.78 | 0.72 | 0.46 | 1.00 | 0.39 | 0.30 |
| Lgals3 | 0.06 | -0.19 | 0.20 | 0.35 | 0.10 | 0.53 | 0.44 | 0.84 | 0.39 | 0.14 | 0.63 | 0.85 | 0.39 | 1.00 | 0.80 |
| Ccn2 | 0.12 | -0.13 | 0.23 | 0.30 | 0.13 | 0.62 | 0.52 | 0.76 | 0.35 | 0.11 | 0.53 | 0.78 | 0.30 | 0.80 | 1.00 |
| Parameters | Klkb1 | Kng1 | F2r | F2 | Il1b | Tnfa | Hmgb1 | Ccl2 | Ccl3 | Adgre1 | Ly6c | Ly6g | Mpo | Lgals3 | Ccn2 |
| --- | --- | --- | --- | --- | --- | --- | --- | --- | --- | --- | --- | --- | --- | --- | --- |
| Klkb1 | | 0.000 | 0.000 | 0.000 | 0.000 | 0.226 | 0.000 | 0.112 | 0.000 | 0.000 | 0.000 | 0.523 | 0.000 | 0.752 | 0.557 |
| Kng1 | 0.000 | | 0.000 | 0.002 | 0.000 | 0.012 | 0.019 | 0.987 | 0.001 | 0.000 | 0.016 | 0.908 | 0.015 | 0.346 | 0.498 |
| F2r | 0.000 | 0.000 | | 0.000 | 0.000 | 0.344 | 0.000 | 0.011 | 0.000 | 0.000 | 0.000 | 0.289 | 0.001 | 0.316 | 0.239 |
| F2 | 0.000 | 0.002 | 0.000 | | 0.000 | 0.690 | 0.000 | 0.003 | 0.001 | 0.000 | 0.000 | 0.045 | 0.000 | 0.069 | 0.125 |
| Il1b | 0.000 | 0.000 | 0.000 | 0.000 | | 0.304 | 0.000 | 0.037 | 0.000 | 0.000 | 0.000 | 0.350 | 0.000 | 0.624 | 0.507 |
| Tnfa | 0.226 | 0.012 | 0.344 | 0.690 | 0.304 | | 0.295 | 0.002 | 0.636 | 0.487 | 0.267 | 0.007 | 0.721 | 0.003 | 0.000 |
| Hmgb1 | 0.000 | 0.019 | 0.000 | 0.000 | 0.000 | 0.295 | | 0.000 | 0.000 | 0.000 | 0.000 | 0.035 | 0.000 | 0.019 | 0.005 |
| Ccl2 | 0.112 | 0.987 | 0.011 | 0.003 | 0.037 | 0.002 | 0.000 | | 0.007 | 0.020 | 0.000 | 0.000 | 0.004 | 0.000 | 0.000 |
| Ccl3 | 0.000 | 0.001 | 0.000 | 0.001 | 0.000 | 0.636 | 0.000 | 0.007 | | 0.000 | 0.000 | 0.034 | 0.003 | 0.043 | 0.070 |
| Adgre1 | 0.000 | 0.000 | 0.000 | 0.000 | 0.000 | 0.487 | 0.000 | 0.020 | 0.000 | | 0.000 | 0.361 | 0.000 | 0.489 | 0.583 |
| Ly6c | 0.000 | 0.016 | 0.000 | 0.000 | 0.000 | 0.267 | 0.000 | 0.000 | 0.000 | 0.000 | | 0.002 | 0.000 | 0.000 | 0.003 |
| Ly6g | 0.523 | 0.908 | 0.289 | 0.045 | 0.350 | 0.007 | 0.035 | 0.000 | 0.034 | 0.361 | 0.002 | | 0.013 | 0.000 | 0.000 |
| Mpo | 0.000 | 0.015 | 0.001 | 0.000 | 0.000 | 0.721 | 0.000 | 0.004 | 0.003 | 0.000 | 0.000 | 0.013 | | 0.042 | 0.126 |
| Lgals3 | 0.752 | 0.346 | 0.316 | 0.069 | 0.624 | 0.003 | 0.019 | 0.000 | 0.043 | 0.489 | 0.000 | 0.000 | 0.042 | | 0.000 |
| Ccn2 | 0.557 | 0.498 | 0.239 | 0.125 | 0.507 | 0.000 | 0.005 | 0.000 | 0.070 | 0.583 | 0.003 | 0.000 | 0.126 | 0.000 | |
B
Supplementary figure 1: Correlation analysis of association between all the assessed genes after acute liver injury.
Tables showing r (A) and p (B) values of the correlation.

## Slide 3
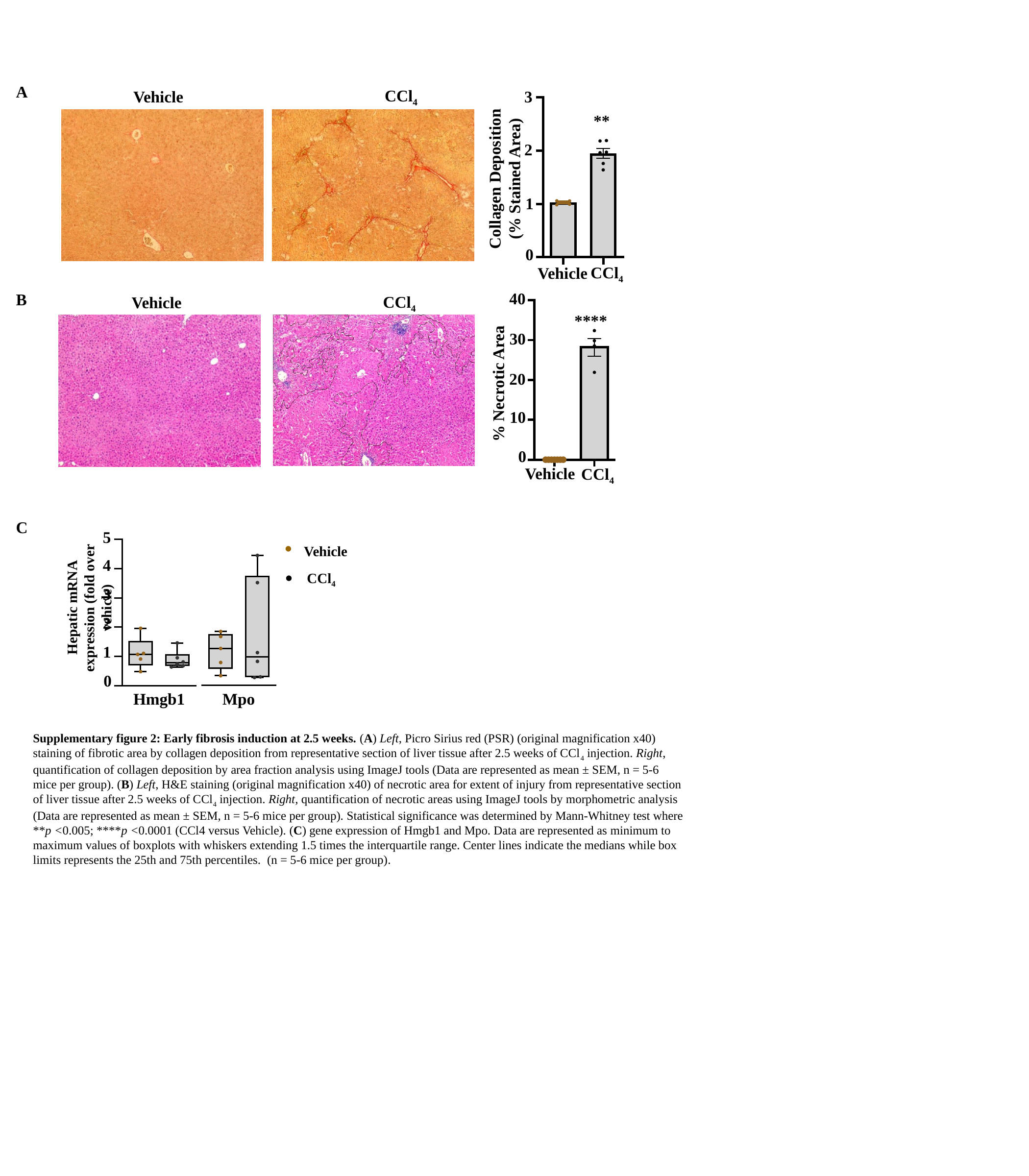

A
CCl4
Vehicle
3
**
2
Collagen Deposition (% Stained Area)
1
0
CCl4
Vehicle
40
B
CCl4
Vehicle
****
30
20
% Necrotic Area
10
0
Vehicle
CCl4
C
5
Vehicle
4
CCl4
3
Hepatic mRNA expression (fold over vehicle)
2
1
0
Hmgb1
Mpo
Supplementary figure 2: Early fibrosis induction at 2.5 weeks. (A) Left, Picro Sirius red (PSR) (original magnification x40) staining of fibrotic area by collagen deposition from representative section of liver tissue after 2.5 weeks of CCl4 injection. Right, quantification of collagen deposition by area fraction analysis using ImageJ tools (Data are represented as mean ± SEM, n = 5-6 mice per group). (B) Left, H&E staining (original magnification x40) of necrotic area for extent of injury from representative section of liver tissue after 2.5 weeks of CCl4 injection. Right, quantification of necrotic areas using ImageJ tools by morphometric analysis (Data are represented as mean ± SEM, n = 5-6 mice per group). Statistical significance was determined by Mann-Whitney test where **p <0.005; ****p <0.0001 (CCl4 versus Vehicle). (C) gene expression of Hmgb1 and Mpo. Data are represented as minimum to maximum values of boxplots with whiskers extending 1.5 times the interquartile range. Center lines indicate the medians while box limits represents the 25th and 75th percentiles.  (n = 5-6 mice per group).

## Slide 4
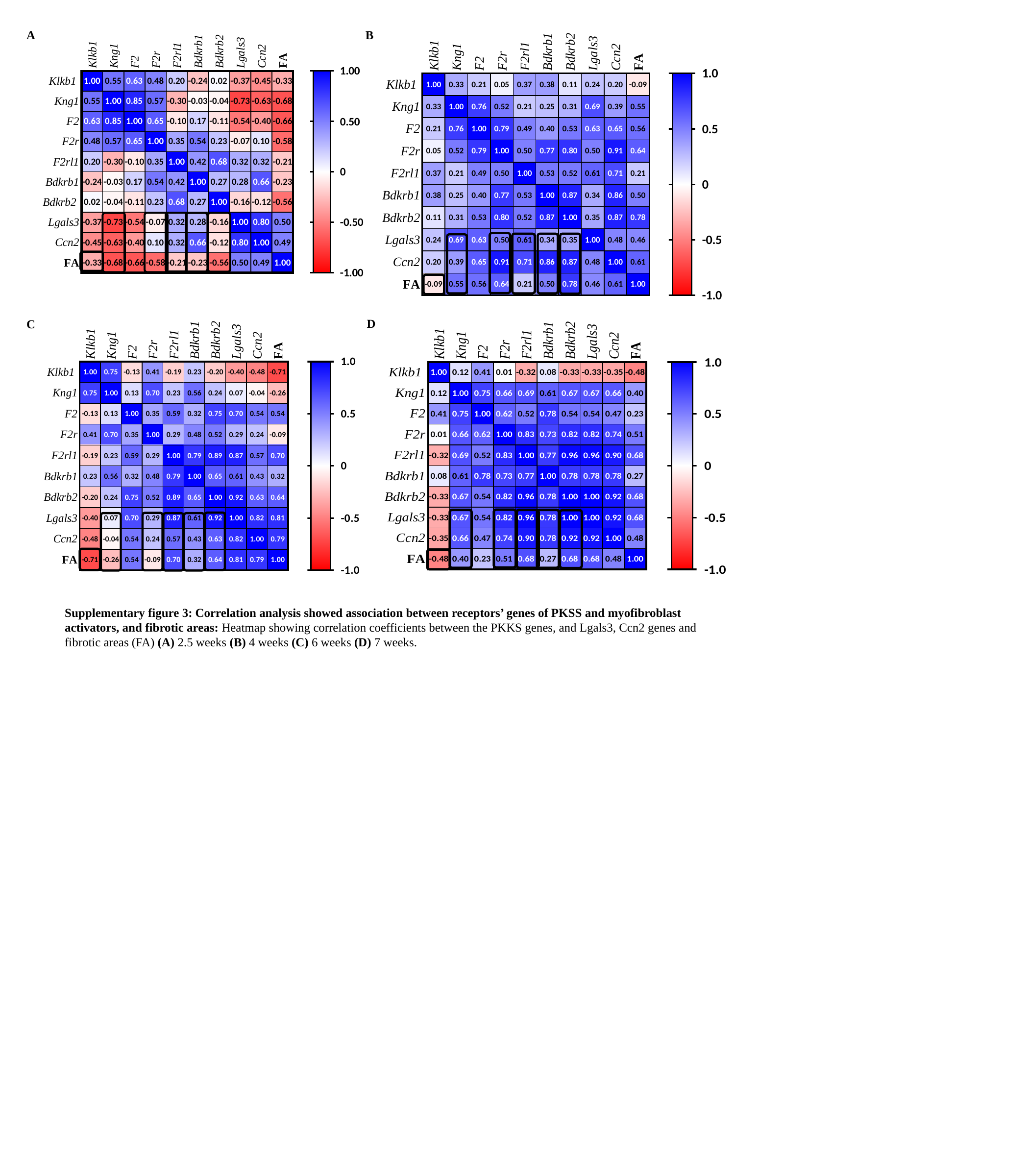

B
A
D
C
Supplementary figure 3: Correlation analysis showed association between receptors’ genes of PKSS and myofibroblast activators, and fibrotic areas: Heatmap showing correlation coefficients between the PKKS genes, and Lgals3, Ccn2 genes and fibrotic areas (FA) (A) 2.5 weeks (B) 4 weeks (C) 6 weeks (D) 7 weeks.

## Slide 5
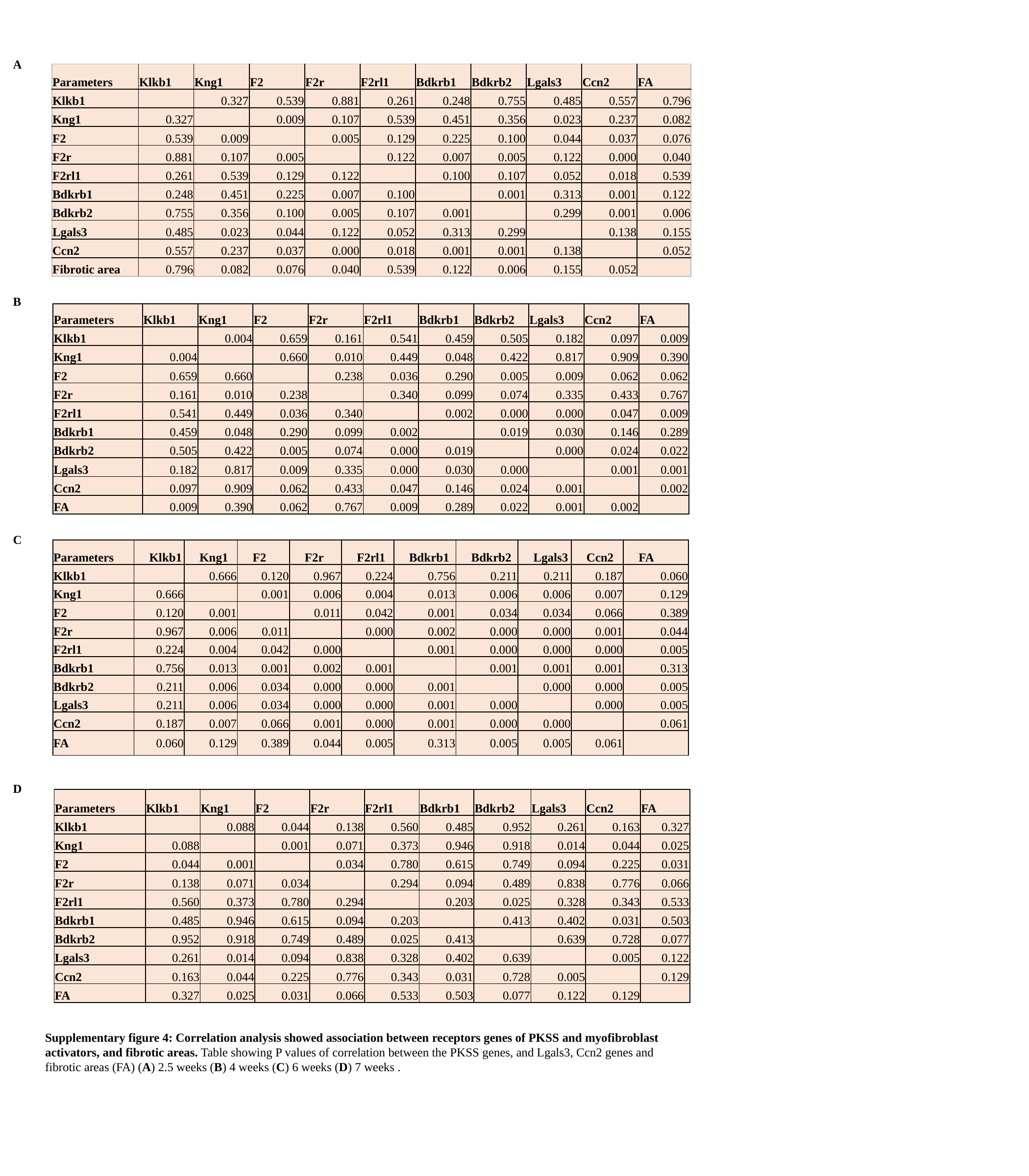

A
| Parameters | Klkb1 | Kng1 | F2 | F2r | F2rl1 | Bdkrb1 | Bdkrb2 | Lgals3 | Ccn2 | FA |
| --- | --- | --- | --- | --- | --- | --- | --- | --- | --- | --- |
| Klkb1 | | 0.327 | 0.539 | 0.881 | 0.261 | 0.248 | 0.755 | 0.485 | 0.557 | 0.796 |
| Kng1 | 0.327 | | 0.009 | 0.107 | 0.539 | 0.451 | 0.356 | 0.023 | 0.237 | 0.082 |
| F2 | 0.539 | 0.009 | | 0.005 | 0.129 | 0.225 | 0.100 | 0.044 | 0.037 | 0.076 |
| F2r | 0.881 | 0.107 | 0.005 | | 0.122 | 0.007 | 0.005 | 0.122 | 0.000 | 0.040 |
| F2rl1 | 0.261 | 0.539 | 0.129 | 0.122 | | 0.100 | 0.107 | 0.052 | 0.018 | 0.539 |
| Bdkrb1 | 0.248 | 0.451 | 0.225 | 0.007 | 0.100 | | 0.001 | 0.313 | 0.001 | 0.122 |
| Bdkrb2 | 0.755 | 0.356 | 0.100 | 0.005 | 0.107 | 0.001 | | 0.299 | 0.001 | 0.006 |
| Lgals3 | 0.485 | 0.023 | 0.044 | 0.122 | 0.052 | 0.313 | 0.299 | | 0.138 | 0.155 |
| Ccn2 | 0.557 | 0.237 | 0.037 | 0.000 | 0.018 | 0.001 | 0.001 | 0.138 | | 0.052 |
| Fibrotic area | 0.796 | 0.082 | 0.076 | 0.040 | 0.539 | 0.122 | 0.006 | 0.155 | 0.052 | |
B
| Parameters | Klkb1 | Kng1 | F2 | F2r | F2rl1 | Bdkrb1 | Bdkrb2 | Lgals3 | Ccn2 | FA |
| --- | --- | --- | --- | --- | --- | --- | --- | --- | --- | --- |
| Klkb1 | | 0.004 | 0.659 | 0.161 | 0.541 | 0.459 | 0.505 | 0.182 | 0.097 | 0.009 |
| Kng1 | 0.004 | | 0.660 | 0.010 | 0.449 | 0.048 | 0.422 | 0.817 | 0.909 | 0.390 |
| F2 | 0.659 | 0.660 | | 0.238 | 0.036 | 0.290 | 0.005 | 0.009 | 0.062 | 0.062 |
| F2r | 0.161 | 0.010 | 0.238 | | 0.340 | 0.099 | 0.074 | 0.335 | 0.433 | 0.767 |
| F2rl1 | 0.541 | 0.449 | 0.036 | 0.340 | | 0.002 | 0.000 | 0.000 | 0.047 | 0.009 |
| Bdkrb1 | 0.459 | 0.048 | 0.290 | 0.099 | 0.002 | | 0.019 | 0.030 | 0.146 | 0.289 |
| Bdkrb2 | 0.505 | 0.422 | 0.005 | 0.074 | 0.000 | 0.019 | | 0.000 | 0.024 | 0.022 |
| Lgals3 | 0.182 | 0.817 | 0.009 | 0.335 | 0.000 | 0.030 | 0.000 | | 0.001 | 0.001 |
| Ccn2 | 0.097 | 0.909 | 0.062 | 0.433 | 0.047 | 0.146 | 0.024 | 0.001 | | 0.002 |
| FA | 0.009 | 0.390 | 0.062 | 0.767 | 0.009 | 0.289 | 0.022 | 0.001 | 0.002 | |
C
| Parameters | Klkb1 | Kng1 | F2 | F2r | F2rl1 | Bdkrb1 | Bdkrb2 | Lgals3 | Ccn2 | FA |
| --- | --- | --- | --- | --- | --- | --- | --- | --- | --- | --- |
| Klkb1 | | 0.666 | 0.120 | 0.967 | 0.224 | 0.756 | 0.211 | 0.211 | 0.187 | 0.060 |
| Kng1 | 0.666 | | 0.001 | 0.006 | 0.004 | 0.013 | 0.006 | 0.006 | 0.007 | 0.129 |
| F2 | 0.120 | 0.001 | | 0.011 | 0.042 | 0.001 | 0.034 | 0.034 | 0.066 | 0.389 |
| F2r | 0.967 | 0.006 | 0.011 | | 0.000 | 0.002 | 0.000 | 0.000 | 0.001 | 0.044 |
| F2rl1 | 0.224 | 0.004 | 0.042 | 0.000 | | 0.001 | 0.000 | 0.000 | 0.000 | 0.005 |
| Bdkrb1 | 0.756 | 0.013 | 0.001 | 0.002 | 0.001 | | 0.001 | 0.001 | 0.001 | 0.313 |
| Bdkrb2 | 0.211 | 0.006 | 0.034 | 0.000 | 0.000 | 0.001 | | 0.000 | 0.000 | 0.005 |
| Lgals3 | 0.211 | 0.006 | 0.034 | 0.000 | 0.000 | 0.001 | 0.000 | | 0.000 | 0.005 |
| Ccn2 | 0.187 | 0.007 | 0.066 | 0.001 | 0.000 | 0.001 | 0.000 | 0.000 | | 0.061 |
| FA | 0.060 | 0.129 | 0.389 | 0.044 | 0.005 | 0.313 | 0.005 | 0.005 | 0.061 | |
D
| Parameters | Klkb1 | Kng1 | F2 | F2r | F2rl1 | Bdkrb1 | Bdkrb2 | Lgals3 | Ccn2 | FA |
| --- | --- | --- | --- | --- | --- | --- | --- | --- | --- | --- |
| Klkb1 | | 0.088 | 0.044 | 0.138 | 0.560 | 0.485 | 0.952 | 0.261 | 0.163 | 0.327 |
| Kng1 | 0.088 | | 0.001 | 0.071 | 0.373 | 0.946 | 0.918 | 0.014 | 0.044 | 0.025 |
| F2 | 0.044 | 0.001 | | 0.034 | 0.780 | 0.615 | 0.749 | 0.094 | 0.225 | 0.031 |
| F2r | 0.138 | 0.071 | 0.034 | | 0.294 | 0.094 | 0.489 | 0.838 | 0.776 | 0.066 |
| F2rl1 | 0.560 | 0.373 | 0.780 | 0.294 | | 0.203 | 0.025 | 0.328 | 0.343 | 0.533 |
| Bdkrb1 | 0.485 | 0.946 | 0.615 | 0.094 | 0.203 | | 0.413 | 0.402 | 0.031 | 0.503 |
| Bdkrb2 | 0.952 | 0.918 | 0.749 | 0.489 | 0.025 | 0.413 | | 0.639 | 0.728 | 0.077 |
| Lgals3 | 0.261 | 0.014 | 0.094 | 0.838 | 0.328 | 0.402 | 0.639 | | 0.005 | 0.122 |
| Ccn2 | 0.163 | 0.044 | 0.225 | 0.776 | 0.343 | 0.031 | 0.728 | 0.005 | | 0.129 |
| FA | 0.327 | 0.025 | 0.031 | 0.066 | 0.533 | 0.503 | 0.077 | 0.122 | 0.129 | |
Supplementary figure 4: Correlation analysis showed association between receptors genes of PKSS and myofibroblast activators, and fibrotic areas. Table showing P values of correlation between the PKSS genes, and Lgals3, Ccn2 genes and fibrotic areas (FA) (A) 2.5 weeks (B) 4 weeks (C) 6 weeks (D) 7 weeks .
